# A proteomics approach to study mouse long bones: examining baseline differences and mechanical loading-induced bone formation in young-adult and old mice

**DOI:** 10.18632/aging.206131

**Published:** 2024-10-12

**Authors:** Christopher J. Chermside-Scabbo, John T. Shuster, Petra Erdmann-Gilmore, Eric Tycksen, Qiang Zhang, R. Reid Townsend, Matthew J. Silva

**Affiliations:** 1 Department of Orthopaedic Surgery, Washington University School of Medicine, St. Louis, MO 63110, USA; 2 Medical Scientist Training Program, Washington University School of Medicine, St. Louis, MO 63110, USA; 3 Department of Medicine, Proteomics Core, Washington University School of Medicine, St. Louis, MO 63110, USA; 4 Department of Genetics, McDonnell Genome Institute, Washington University School of Medicine, St. Louis, MO 63108, USA; 5 Department of Biomedical Engineering, Washington University, St. Louis, MO 63105, USA

**Keywords:** bone, aging, mechanical loading, proteomics, RNA-seq/transcriptomics

## Abstract

With aging, bone mass declines and the anabolic effects of skeletal loading diminish. While much research has focused on gene transcription, how bone ages and loses its mechanoresponsiveness at the protein level remains unclear. We developed a novel proteomics approach and performed a paired mass spectrometry and RNA-seq analysis on tibias from young-adult (5-month) and old (22-month) mice. We report the first correlation estimate between the bone proteome and transcriptome (Spearman *ρ* = 0.40), which is in line with other tissues but indicates that a relatively low amount of variation in protein levels is explained by the variation in transcript levels. Of 71 shared targets that differed with age, eight were associated with bone mineral density in previous GWAS, including understudied targets Asrgl1 and Timp2. We used complementary RNA *in situ* hybridization to confirm that Asrgl1 and Timp2 had reduced expression in osteoblasts/osteocytes in old bones. We also found evidence for reduced TGF-beta signaling with aging, in particular Tgfb2. Next, we defined proteomic changes following mechanical loading. At the protein level, bone differed more with age than with loading, and aged bone had fewer loading-induced changes. Overall, our findings underscore the need for complementary protein-level assays in skeletal biology research.

## INTRODUCTION

Osteoporosis and age-related fractures remain a significant public health burden [1–4]. In a given year, the number of women who sustain a fracture is greater than the combined number of women diagnosed with incident breast cancer, myocardial infarction, or stroke [5, 6]. Despite this burden, the mechanisms underlying the age-related decline in bone mass remain incompletely understood [7]. A more complete understanding of these age-related changes may lead to new approaches to prevent or reverse osteoporosis.

While unbiased ‘-omics’ approaches such as RNA sequencing (RNA-seq) have revealed much about bone and how it ages [8–11], an understanding of the protein-level changes in aged bone remains limited. Importantly, many age-related changes in other tissues occur post-transcriptionally [12]. Liquid chromatography-mass spectrometry (LC-MS/MS)–based proteomics is a technology that facilitates a comprehensive view of biological systems at the protein level [13]. It has been used with *in vitro* models to extend the understanding of the molecular mechanisms that regulate bone cells [14, 15]. One previous study compared the proteome of exosomes isolated from bones of young and aged mice [16], yet to our knowledge, an evaluation of age-related changes on bulk cortical bone tissue has not been described. In addition, while proteomic analyses of bone samples have been reported [17–20], no study has shown the feasibility of a whole-tissue proteomic analysis of mouse long bones, a ubiquitous model in skeletal biology research. We sought to assess the feasibility of a proteomic analysis of mouse cortical bone, to estimate the correlation between the proteome and transcriptome in bone tissue using LC-MS/MS and RNA-seq on paired limbs from the same mice, and to use proteomics to investigate the age-related changes at the protein-level in mouse cortical bone.

In youth, mechanical loading potently induces bone formation [21, 22], but with aging, this response to mechanical loading declines in both humans [23–25] and rodents [26–30]. However, because the mechanisms driving bone’s age-related decrease in mechanoresponsiveness remain incompletely understood, targeting the involved processes for maximal clinical benefit has been limited. We have previously used RNA-seq to characterize the loading response in young-adult (5-month) and old (22-month) mice [11]. We showed that old mice had less transcriptional activity following loading compared to young-adult mice, and identified a number of targets to pursue to restore the age-related decline in mechanoresponsiveness. Here, we sought to extend these findings by assessing the age-related differences in the response to loading at the protein level.

First, to discover age-related differences at baseline in the proteome and transcriptome, we performed paired proteomics and RNA-seq on tibias of young-adult (5-month) and old (22-month) C57BL/6N female mice not subjected to any interventions. We used these findings to gain an understanding of how the bone proteome relates to the transcriptome in bone and to identify age-related targets. Second, to extend our understanding of the age-related decline in mechanoresponsiveness, we used proteomics to compare the loading responses between tibias of young-adult and old mice following 1 or 5 days of a well-characterized, axial loading protocol.

## RESULTS

### Protein and gene expression in bone correlate moderately

**Figure 1:**
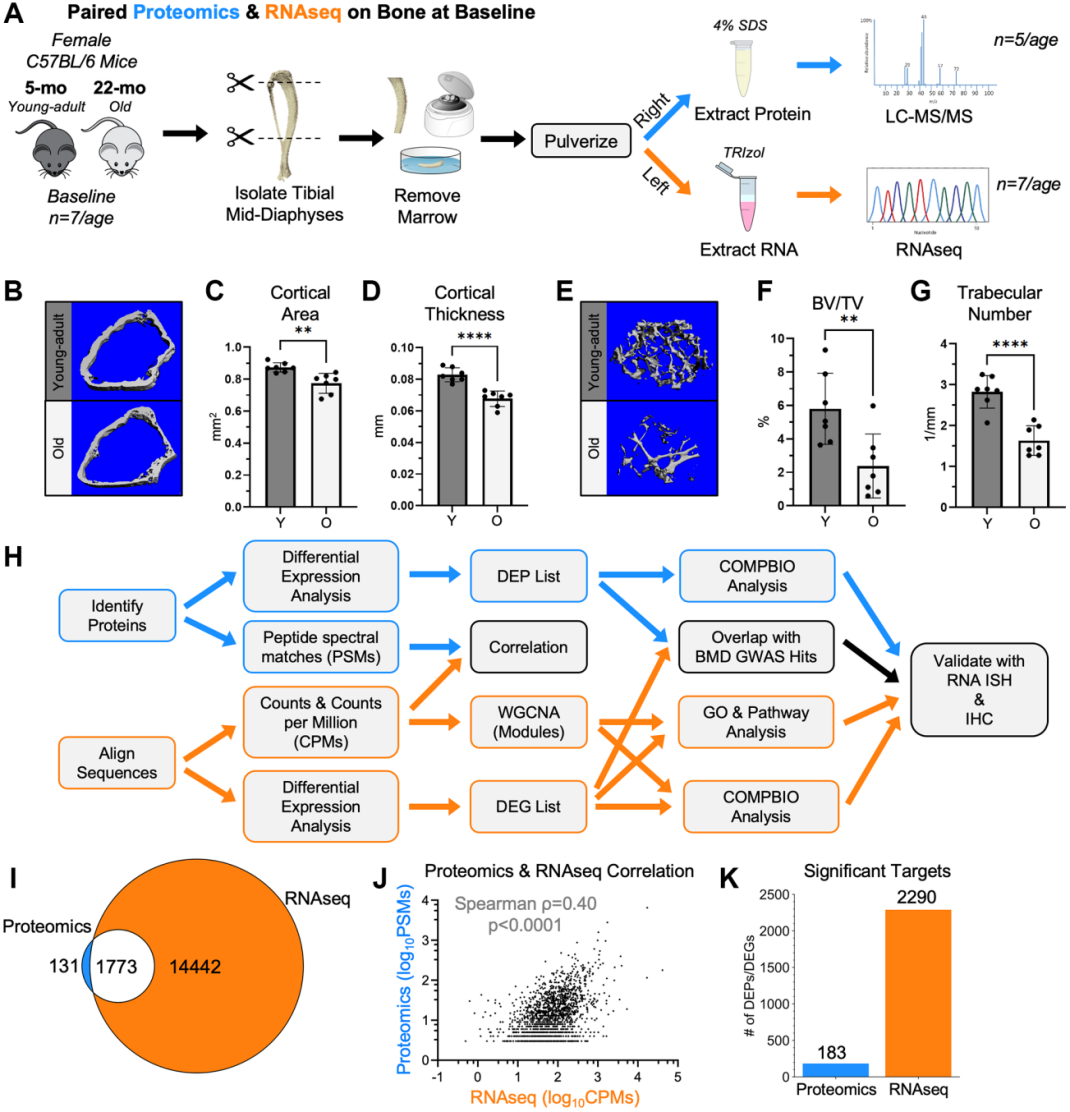
**RNA-seq and proteomics were used to characterize cortical bone from young-adult and old mice at baseline.** (**A**) Untreated 5-month-old (young-adult) and 22-month-old (old) female C57BL/6N mice were sacrificed. Paired right and left tibial mid-diaphyses were isolated, removed of marrow, and snap frozen. From the right tibias, proteins were extracted using 4% SDS. Proteins from 5 tibias per age were analyzed by proteomics using a tandem mass tag (TMT)-11. From the left tibias, RNA was isolated using TRIzol. RNA from 7 tibias per age was sequenced. (**B**) MicroCT of the distal right femurs from these mice confirmed the expected age-related differences in the cortical bone. (**C**, **D**) The distal cortical bone area and cortical thickness were lower with age. (**E**) MicroCT also confirmed age-related changes in the trabecular bone of the distal femur. (**F**, **G**) The bone volume per total volume (BV/TV) and trabecular number were lower with age. (**H**) Proteomics and RNA-seq raw data were analyzed, and differential expression analysis was performed separately. For both methods, a Benjamini-Hochberg-adjusted *p*-value cutoff of 0.05 was used to identify differentially expressed genes (DEGs) and differentially expressed proteins (DEPs). Downstream analyses included correlations, overlaps, weighted gene co-expression network analysis (WGCNA), gene ontology (GO) analysis, pathway analysis, and COMPBIO analysis. (**I**) 93% (1773/1904) of proteomics hits (PSM≥3) were detectable by RNA-seq (non-zero CPM for all samples). (**J**) The abundance of the 1773 targets detected by both proteomics and RNA-seq (after PSM and CPM filtering) were correlated (Spearman). (**K**) Comparing young-adult and old bone at baseline, 183 proteomics targets and 2290 RNA-seq targets met the *p*-value cutoff to be DEPs and DEGs, respectively. Abbreviations: SDS: Sodium dodecyl sulfate; BV/TV: Bone Volume/Total Volume; DEG: Differentially Expressed Gene; DEP: Differentially Expressed Protein; CPMs: Counts per million; PSMs: Peptide spectral matches.

Paired tibias from young-adult and old mice at baseline were analyzed by proteomics and RNA-seq (Figure 1A, 1H). Femurs from these mice exhibited the expected age-related changes in bone morphology (Figure 1B–1G). After filtering, 1903 proteins were detected by proteomics, and 16273 genes were detected by RNA-seq (Supplementary Figure 1). At both the protein and RNA levels, multidimensional scaling (MDS) showed that young-adult samples separated from old samples. Of the detected proteins, 93% (1773/1903) were also detectable by RNA-seq (Figure 1I). Using the average peptide spectral matches (PSMs) and average counts per million (CPMs), the abundance of the 1773 targets detected by both proteomics and RNA-seq were correlated (Figure 1J), as reported [31]. The correlation was moderately positive (Spearman *ρ* = 0.40, *p* < 0.001), consistent with results in other tissues [31, 32]. Differential expression analysis between young-adult and old bone was performed separately for proteomics and RNA-seq. 183 proteins and 2290 genes met the *p*-value cutoff to be age-related differentially expressed proteins (DEPs) and differentially expressed genes (DEGs), respectively (Figure 1K).

### Many of the most significantly up- and downregulated proteins and genes between young-adult and old bone have been associated with bone phenotypes in GWAS

We compiled the top 15 most significantly differentially expressed (by FDR) proteins and genes (both up- and downregulated) between young-adult and old bone at baseline (Table 1). Several of these targets have been associated with phenotypes related to bone [33–49] or aging [50, 51] in genome-wide association studies (GWAS). The only top-15 target identified by both platforms was MMP13, which was more highly expressed in old bone at both the protein and transcript level. MMP13 is critical for osteocyte perilacunar remodeling [52] and maintains cartilage homeostasis [53].

**Table 1:** Top 15 differentially expressed genes and proteins comparing old vs. young-adult at baseline, ranked by false discovery rate (FDR).

	**Proteomics**				**RNA-seq**			
	**Protein**	**FC**	**FDR**	**Relevant GWAS**	**Gene**	**FC**	**FDR**	**Relevant GWAS**
***Upregulated***	CTSS	2.0	4.6E-03	BMD [33]	*Tubb6*	2.0	9.5E-05	−
	PYCARD	1.9	1.2E-02	−	*Pstpip1*	2.0	1.6E-04	Height [36]
	OSTF1	1.8	1.2E-02	Scoliosis [34]	*Tfrc*	1.9	2.2E-04	−
	PYGL	1.8	1.3E-02	−	*H6pd*	1.4	2.6E-04	BMD [36] Height [44]
	SET	1.5	1.3E-02	−	*Dock5*	1.8	2.9E-04	BMD [33] Scoliosis [34]
	**MMP13**	1.6	1.4E-02	−	*Tyrobp*	1.8	3.2E-04	−
	IGHM	1.9	2.0E-02	−	*Pik3ap1*	2.0	3.3E-04	Scoliosis [34] Longevity [50]
	IPO5	1.9	2.0E-02	BMD [35]	*Cebpb*	1.8	3.3E-04	−
	HMGCL	1.8	2.0E-02	−	*Ptpn22*	2.0	3.3E-04	−
	CD36	1.8	2.0E-02	Height [36]	*Galnt6*	2.4	3.4E-04	−
	G6PC3	1.8	2.0E-02	−	*H2-k1*	1.6	3.9E-04	(HLA-A) BMD [45] Height [42]
	DYSF	1.7	2.0E-02	BMD [36]	*Mapkapk2*	1.5	4.1E-04	−
	ATP6V1C1	1.7	2.0E-02	−	***Mmp13***	2.4	4.2E-04	−
	BTF3	1.6	2.0E-02	BMD [33]	*H2-d1*	1.6	4.2E-04	(HLA-A) BMD [45] Height [42]
	ATP6V1B2	1.6	2.0E-02	−	*Acp5*	2.0	4.4E-04	−
***Downregulated***	SOST	−2.3	8.3E-04	BMD [37, 38] Fracture [39]	*Hdac9*	−3.2	2.7E-06	Height [36]
	BASP1	−1.9	1.8E-03	BMC [35]	*Ltbp1*	−2.0	2.3E-05	Height [46]
	CTHRC1	−1.8	4.4E-03	−	*Magi2*	−2.8	2.3E-05	Height [47]
	IGSF8	−2.2	4.4E-03	−	*Ndufa4l2*	−3.0	2.3E-05	−
	CC194	−2.0	4.4E-03	Height [36]	*Chrdl1*	−3.0	2.9E-05	−
	EFEMP1	−1.7	4.4E-03	Height [40] Skin aging [51]	*Rab27b*	−2.1	3.5E-05	Height [41]
	EFEMP2	−1.7	4.6E-03	Height [41]	*Sytl2*	−2.2	3.7E-05	Height [48]
	COL3A1	−1.8	4.6E-03	−	*Tcf7l2*	−2.1	5.7E-05	BMD [45] Longevity [137] Height [36]
	COL5A1	−1.5	4.6E-03	−	*Smad9*	−2.8	5.7E-05	BMD [45] Height [36]
	NIT2	−1.6	4.6E-03	−	*Ism1*	−3.3	5.7E-05	Height [36]
	MBL1	−1.7	4.6E-03	(MBL2) BMD [39]	*Arnt2*	−3.4	5.7E-05	−
	CD44	−1.7	4.6E-03	−	*Pip4kaA*	−2.2	6.1E-05	Height [49]
	TNN	−2.1	5.0E-03	−	*Sdc3*	−2.2	6.1E-05	−
	S100A10	−1.9	5.5E-03	−	*Adamts17*	−3.4	6.1E-05	Height [40]
	COL11A1	−1.6	5.5E-03	BMD [38] Height [42] Bone Size [43]	*Olfml2a*	−2.3	6.7E-05	Height [36]

FC: Linear fold-change (old relative to young-adult). Bold denotes shared between proteomics and RNA-seq. Brackets denote ortholog.

The most significantly upregulated protein in old bone at baseline was CTSS, a cysteine protease known as cathepsin S that regulates extracellular matrix (ECM) remodeling and antigen presentation. CTSS interacts with osteocalcin (Bglap) and has been shown to control osteoblast differentiation and bone turnover [54]. PYCARD, which promotes apoptosis and inflammation, and OSTF1, which is known as osteoclast stimulating factor 1 and directly induces osteoclast differentiation and bone resorption [55], were almost two-fold higher in old bone. Additional proteins that were higher in old bone include IPO5, an important nuclear transport receptor; CD36, which is important for both osteoblast [56] and osteoclast [57] function; DYSF, which is known to act as a calcium sensor in muscle to facilitate membrane repair [58, 59]; and BTF3, which plays a role in c-Jun transcriptional activity.

The most significantly downregulated protein in old bone at baseline was SOST, commonly called sclerostin. SOST is a well-known Wnt antagonist in bone that is highly expressed by mature osteocytes and is the target of the most recently approved osteoporosis drug romozosumab [60]. BASP1 is also lower in old bone, and although little is known about BASP1 in the context of bone, it was detected in a proteomic profiling of osteoblast differentiation [61]. COL5A1, a target of TGF-beta in bone [62], and COL11A1, which regulates bone microarchitecture during development [63], are both lower in old bone. Four other proteins that were lower in old bone included: CD44, which inhibits inflammatory bone loss [64]; MBL1, a mannose binding lectin that may suppress osteoclastogenesis [65]; TNN, the murine version of tenascin W [66] that is induced in bone cells by loading and Wnt signaling [67]; and S100A1, a calcium binder that is important in mineralization [68].

The most significantly upregulated genes in old bone at baseline play a role in osteoclastic resorption, including *Tubb6* [69], *Pstpip1* [70], and *Dock5* [71, 72]. *Tyrob* affects the myeloid lineage (precursors of osteoclasts) and has been linked to a human disease involving bone cysts [73, 74]. *Cebpb* is known to be important in osteoblast differentiation [75]. *H2-k1* and *H2-d1* are orthologs of the human HLA-A gene, and while HLA-A is expressed by osteoblast-lineage cells [76], the higher expression may reflect more inflammation and immune cell infiltration in older bone [77]. *H6pd*, of the pentose phosphate shunt, and *Pik3ap1*, a player in B-cell development, were also upregulated in old bone. Last, *Acp5*, better known as tartrate-resistant acid phosphatase (TRAP), was also higher in old bone. TRAP is expressed by bone-resorbing osteoclasts and is also expressed by osteocytes during perilacunar remodeling [78].

The most significantly downregulated gene in old bone at baseline was *Hdac9*, an inhibitor of osteoclastogenesis [79]. A number of other downregulated genes are related to TGF-beta or bone morphogenetic protein (BMP) signaling. *Ltbp1*, known as latent TGF-beta binding protein 1, directly modulates TGF-beta activity and is substrate of matrix metalloproteinases (MMPs) [80]. The lower expression of *Ltbp1* may relate to lower levels of the identified BMP regulators *Smad9* [81], *Chrdl1* [82], and *Adamts17* [83]. *Nduf4l2*, which has been suggested to be important for the metabolic transition of osteoblasts into osteocytes, was lower in old bone [84]. Similar to findings at the protein level, *Sost* was also a downregulated DEG (FC: -3.9, FDR: 9.5E-05). Other genes more lowly expressed in old bone included *Magi2* and *Pip4k2a*, which may play a role in PI3K-AKT signaling, and *Tcf7l2,* which is a Wnt effector transcription factor [85].

### Integrated analysis of GWAS BMD hits with baseline proteomics and RNA-seq from young-adult and old bone identified eight targets including Asrgl1 and Timp2

**Figure 2:**
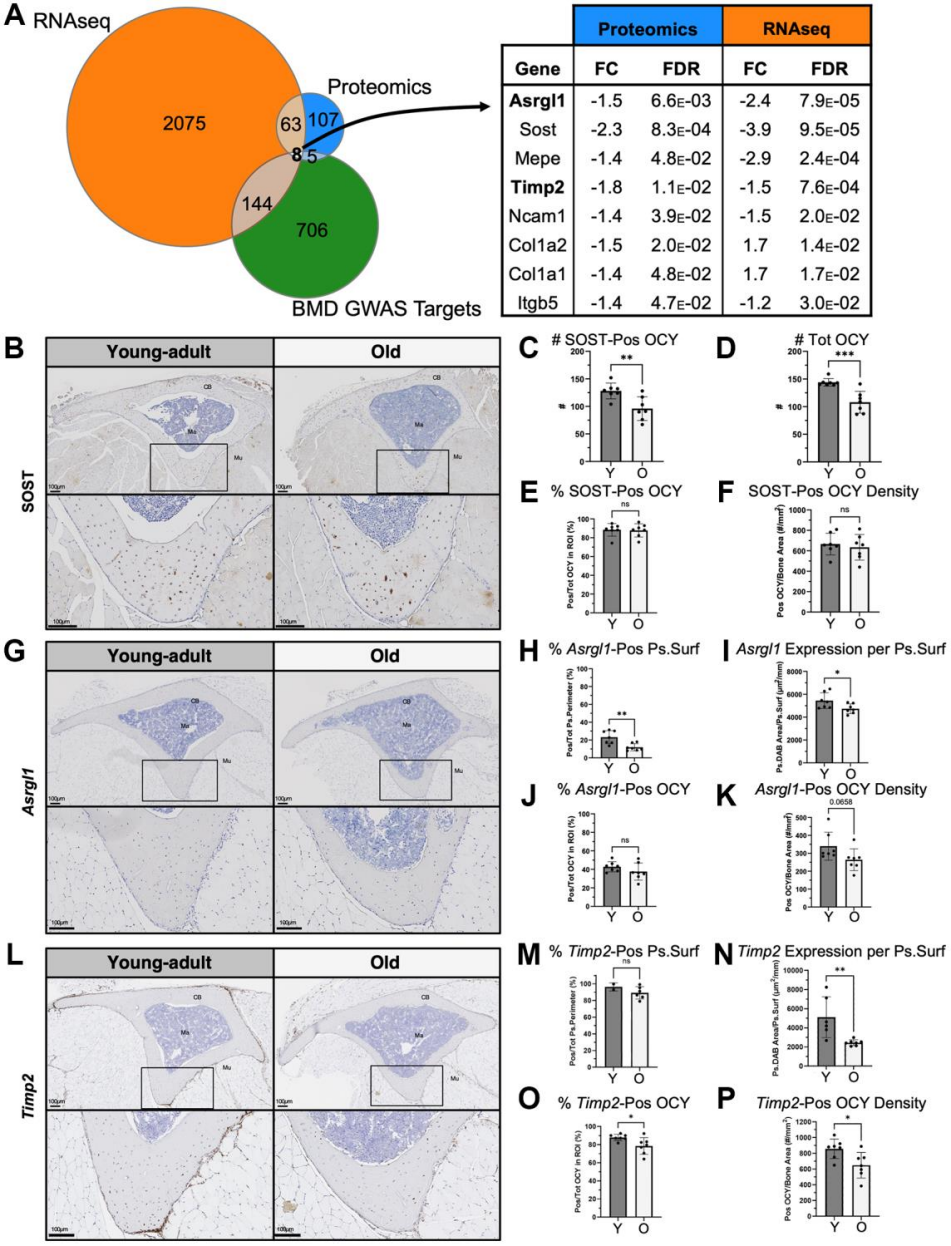
**Integrated analysis of GWAS BMD hits with baseline proteomics and RNA-seq identified eight targets including Asrgl1 and Timp2.** (**A**) Intersection of baseline proteomics DEPs, RNA-seq DEGs, and a GWAS database [39] revealed 8 targets. Of these, Sost, Asrgl1 and Timp2 were further evaluated histologically. (**B**) SOST expression was validated using IHC in tibias of young-adult and old mice (transverse sections cut at the proximal diaphysis). SOST expression was restricted to osteocytes. (**C**) Fewer SOST-positive and (**D**) total osteocytes (per 600 mm x 600 mm ROI) were present in old bone. (**E**) The percentage of SOST-positive osteocytes and (**F**) the areal density of SOST-positive osteocytes were not lower in old bone. (**G**) *Asrgl1* expression was characterized using RNA ISH. (**H**) Most of the periosteal surface was covered by cells expressing *Asrgl1*, and this percentage of cells decreased with age. (**I**) Periosteal *Asrgl1* expression was lower in old bone. (**J**) Slightly less than half of osteocytes (OCY) within cortical bone expressed *Asrgl1*, and this percentage of OCY was not lower in old bone. (**K**) The areal density of Asrgl1-positive osteocytes trended to be lower in old bone but did not reach significance. (**L**) *Timp2* expression was characterized using RNA ISH. (**M**) Most of the periosteal surface was covered by cells expressing *Timp2*, and this percentage of cells trended to decrease with age. (**N**) Periosteal *Timp2* expression was two times lower in old bone. (**O**) Most osteocytes within cortical bone expressed *Timp2*, and this percentage of OCY decreased with age. (**P**) The areal density of *Timp2*-positive osteocytes decreased with age. Abbreviations: OCY: Osteocyte; CB: Cortical bone; Ma: Marrow; Mu: Muscle; Pos: Positive; Ps: Periosteal; Surf: Surface). Data shown as mean +/−SD. *p*-values calculated by unpaired, 2-tailed *t*-test; *n* = 6–7 mice per age.

Between proteomics and RNA-seq, 71 shared targets were differentially expressed between bones from young-adult and old mice at baseline. To further narrow these candidates to those most relevant to human disease, we intersected them with the hits identified in a recent GWAS that identified genetic determinants of BMD [39], resulting in a list of eight targets (Figure 2A). These included the well-known bone factors Sost, Col1a1, Col1a2, and Mepe, as well as Ncam1 [86] and Itgb5 [87, 88] which have also been studied in the context of bone. We also identified two targets Asrgl1 (Asparaginase and isoaspartyl peptidase 1) and Timp2 (Tissue inhibitor of metalloproteinases 2) that have been less described in the context of bone.

To validate our proteomics findings, we assessed SOST expression histologically at the protein level in a separate set of mice. As expected, SOST expression was restricted to osteocytes, particularly the more mature osteocytes further from the surface (Figure 2B). Fewer SOST-positive and total osteocytes per transverse section were present in old bone (Figure 2C, 2D), consistent with the measured lower SOST at the protein level. Contrary to our expectations, the percentage of SOST-positive osteocytes and the areal density of SOST-positive osteocytes was not lower in old bone (Figure 2E, 2F), suggesting that the decrease in measured SOST at the protein level is due to the lower absolute number of osteocytes.

To better characterize Asrgl1 and Timp2, we used RNA ISH to confirm mRNA expression in bone cells and characterize the expression pattern in young-adult and old bone. Periosteal *Asrgl1*-expressing cells cover less than a quarter (23%) of the surface in young-adult bone, and this percentage is lower in old bone (12%, *p* = 0.004, Figure 2G, 2H). Consistent with this, the total periosteal surface expression of *Asrgl1* (based on DAB-positive area) decreased with age (young: 5440, old: 4735 µm^2^/mm, ^*^p = 0.040, Figure 2I). Slightly less than half of osteocytes also express *Asrgl1* in young-adult bone (43%), and this percentage of positive osteocytes was not significantly lower with age (38%, *p* = 0.22, Figure 2J). The areal density of* Asrgl1*-positive osteocytes trended to be lower but did not reach significance (340 vs. 264 cells/mm^2^, *p* = 0.066, Figure 2K). While *Asrgl1* expression is detected in fewer than one half of bone cells, *Timp2* is expressed by most of the cells on the periosteal surface in young-adult and old bone (96% and 89%, respectively; Figure 2L, 2M). The total periosteal surface expression of *Timp2* was decreased by half with age (young: 5115 vs. old: 2446 µm^2^/mm, *p* = 0.007, Figure 2N). Most osteocytes in young-adult bone also express *Timp2* (88%), but this percentage decreases with age (76%, *p* = 0.025, Figure 2O), as does the areal density of* Timp2*-positive (858 vs. 649 cells/mm^2^, *p* = 0.019, Figure 2P). In summary, we observed reduced expression of *Asrgl1* and *Timp2* in bones of old mice using three methods—proteomics, RNA-seq, and RNA ISH.

### An age-related module indicating baseline differences in TGF-beta and Wnt signaling was identified through co-expression analysis of the RNA-seq data

**Figure 3:**
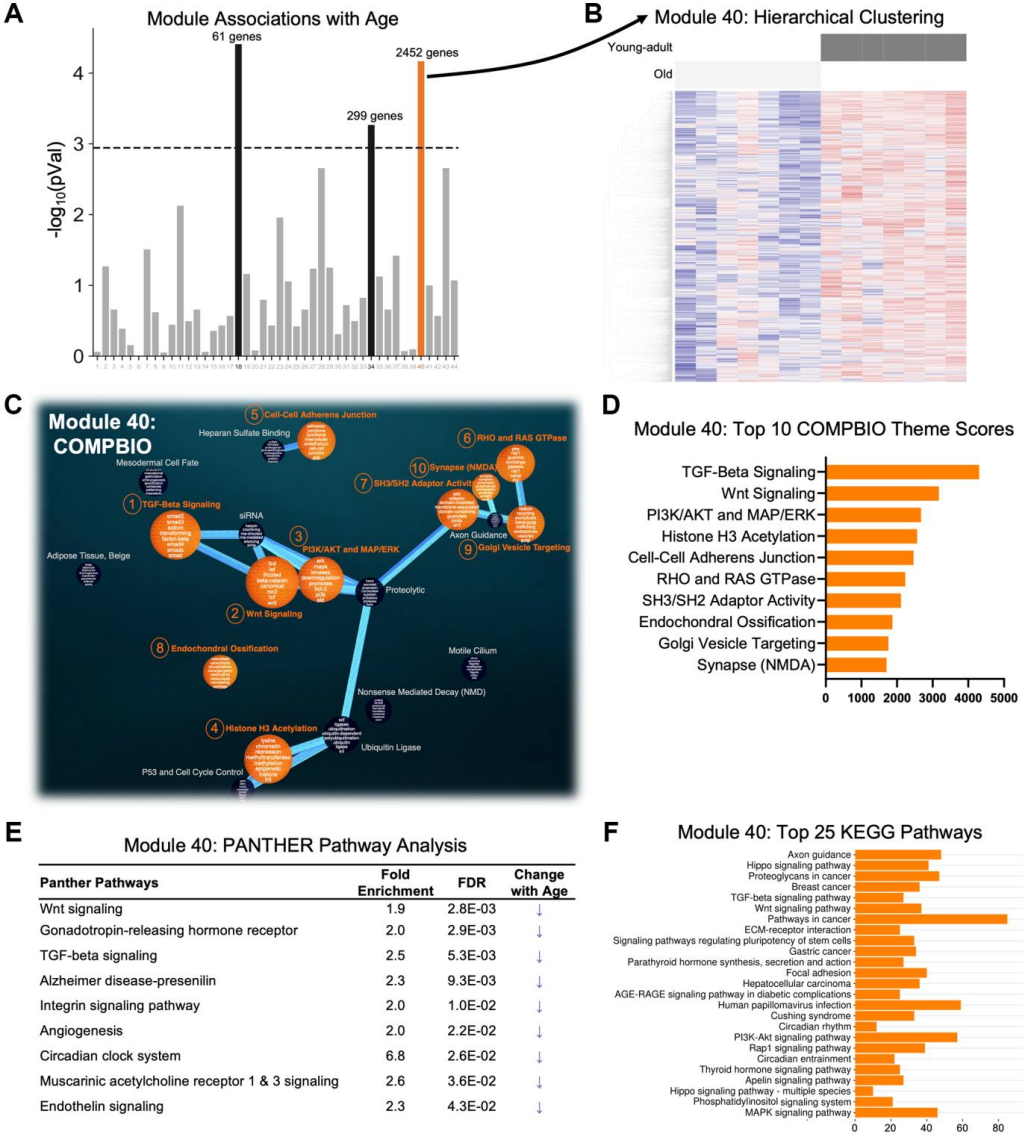
**A single age-related module with perfect separation was identified using weighted gene co-expression network analysis (WGCNA) of the RNA-seq data.** (**A**) Gene counts across all samples were clustered into 44 modules and correlated with age status. Three modules (black and orange) were significantly different between ages using a Bonferroni correction (*p* = 0.0011 = 0.05/44). (**B**) Only Module 40 (orange) displayed perfect separation using hierarchical clustering. (**C**, **D**) COMPBIO analysis of the subset of 1055 genes from Module 40 that had an FDR <0.05 between young-adult and old samples identified TGF-beta signaling and Wnt signaling as the top themes. (**E**, **F**) PANTHER Pathway and KEGG analyses of all 2452 genes in Module 40 also revealed Wnt signaling and TGF-beta signaling, among other pathways. Examination of the fold-changes of the genes from the PANTHER analysis showed that all 9 pathways are reduced with aging. Abbreviation: FE: fold-enrichment.

A co-expression network analysis (WGCNA) of the RNA-seq data identified 44 unique co-expression modules (Figure 3A). Three modules had a significant association with age, but only Module 40, with 2452 genes, correctly separated all young-adult and old samples by hierarchical clustering (Figure 3B and Supplementary Figure 2). We then used COMPBIO to analyze the genes in Module 40, limiting the analysis to the 1055 genes that had an FDR <0.05 in the DEG analysis (young-adult vs. old). Within Module 40, we identified numerous themes that were enriched. The top three themes were TGF-beta signaling, Wnt signaling, and Pi3K/AKT and MAP/ERK, and notably these themes were interconnected in the network (Figure 3C). Other top themes included histone H3 acetylation, cell-cell adherens junctions, and endochondral ossification (Figure 3D). PANTHER pathway analysis of all 2452 genes reinforced differences in Wnt signaling and TGF-beta signaling (Figure 3E). It also identified other pathways including gonadotropin-releasing hormone receptor, Alzheimer disease-presenilin, integrin signaling pathway, and angiogenesis. The genes driving this enrichment were examined for all nine pathways, and nearly all of the genes were more lowly expressed in old bone, suggesting that these pathways are less active in aged bones. KEGG analysis of all 2452 also identified differences in TGF-beta signaling and Wnt signaling but further identified differences that included: axon guidance; hippo signaling; ECM-receptor interaction; parathyroid hormone synthesis, secretion, and action; and AGE/RAGE signaling pathway in diabetic complications (Figure 3F). The diversity of pathways identified in this age-related module underscores the complexity of aging processes occurring simultaneously in bone.

### Baseline age-related differences in ECM/MMPs and TGF-beta signaling were identified in both the proteome and transcriptome

**Figure 4:**
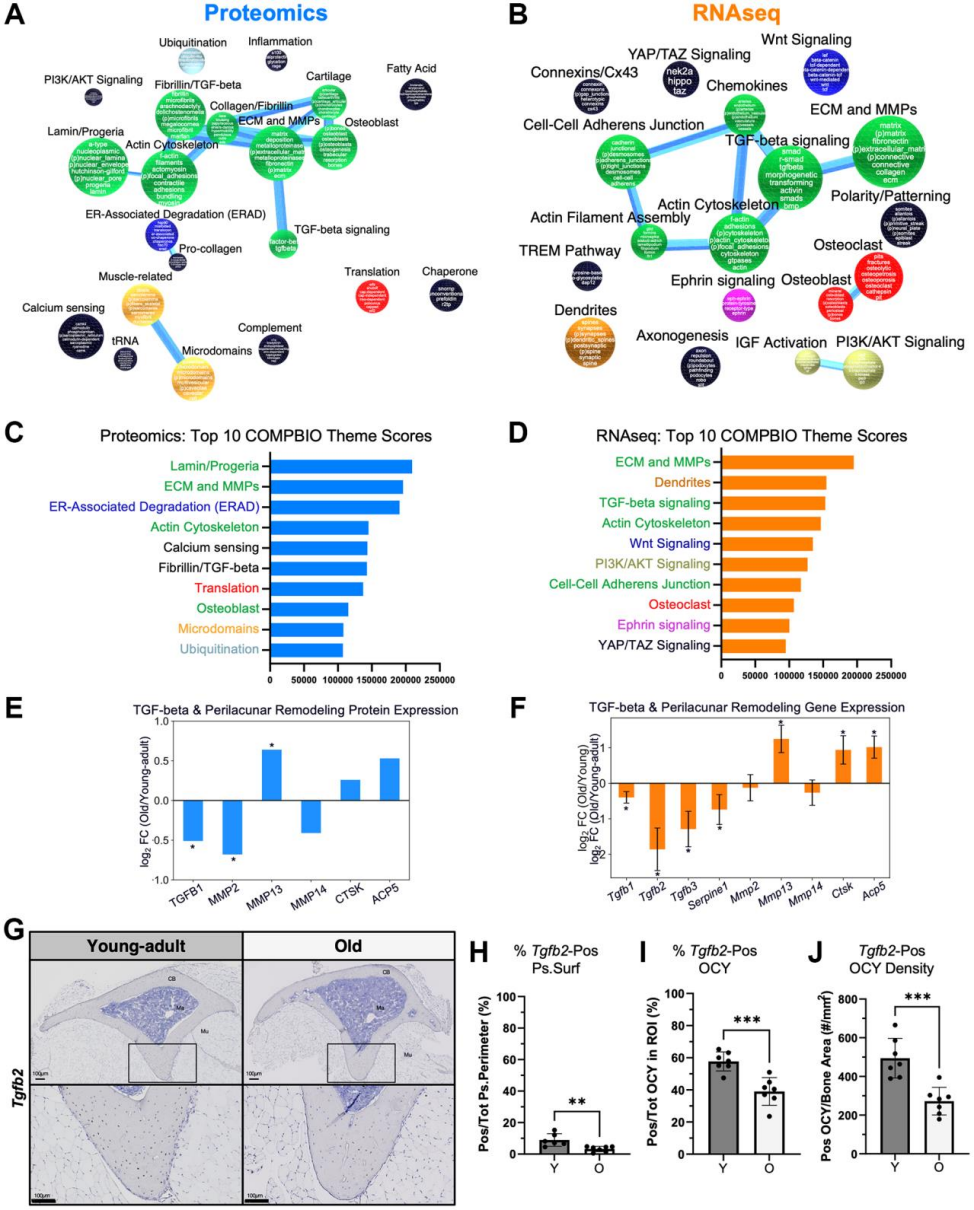
**Baseline age-related differences in ECM/MMPs and TGF-beta signaling were detected in both the proteome and transcriptome.** COMPBIO analysis of (**A**) the proteome and (**B**) the transcriptome indicated baseline changes in ECM/MMPs. (**C**) At the protein level, the top 10 themes included Lamin/Progeria, ECM/MMPs, and ER-Associated Degradation. (**D**) At the RNA level, the top 10 themes included ECM/MMPs, Dendrites, and TGF-beta signaling. Examination of age-related differences in individual (**E**) proteins and (**F**) genes related to TGF-beta signaling and perilacunar remodeling (PLR) showed reduced TGF-beta levels but higher Mmp13. (**G**) RNA ISH for *Tgfb2* showed predominant expression in osteocytes. (**H**) Periosteal *Tgfb2* expression was minimal in young-adult bone and even lower in old bone. (**I**) Over half of osteocytes (OCY) within cortical bone expressed *Tgfb2*, and this percentage was lower in old bone. (**J**) The areal density of *Tgfb2*-positive osteocytes was about half in old versus young bone.

To investigate the age-related processes by both proteomics and transcriptomics, we used COMPBIO to analyze the top 500 (by *p*-value) proteins (Figure 4A) and genes (Figure 4B) that were differentially expressed at baseline between old and young-adult bone. At the protein level, the top themes were nuclear lamin/progeria, ECM/MMPs, and ER-associated degradation (ERAD) (Figure 4C). The most robustly interconnected group, which contained ECM/MMP, also included themes related to actin cytoskeleton, osteoblasts, and TGF-beta signaling. At the RNA level, the top themes were ECM/MMPs, dendrites, and TGF-beta-signaling (Figure 4D). Again, the ECM/MMP-containing group was the most robustly interconnected and additionally included actin cytoskeleton and cell-cell adherens junctions. Notably, only proteomics detected age-related differences related to nuclear lamin/progeria, ERAD, ubiquitination, and chaperone proteins.

Collectively, the most prominent signals involved ECM/MMPs and TGF-beta signaling. Previously, osteocyte-intrinsic TGF-beta signaling has been shown to control perilacunar remodeling (PLR) [89], an important process for maintaining bone quality and fracture resistance [52]. Specifically, TGF-beta signaling is thought to control the expression of MMPs and other matrix-remodeling enzymes such as cathepsin K (CTSK) and tartrate resistant acid phosphatase (ACP5). Therefore, we examined baseline changes in PLR factors in our data. By proteomics, TGF-beta 1 (TGFB1) was the only TGF-beta type detectable, and it was two times lower in old bone (Figure 4E). MMP2 was significantly lower in old bone while MMP13 expression was significantly higher. Other proteins related to perilacunar remodeling such as CTSK and ACP5 trended higher in old bone but did not reach significance. At the transcript level, all three TGF-beta types were detected, and in young-adult bone, *Tgfb1* and *Tgfb2* were the most highly expressed at baseline (Supplementary Figure 3). In old bone, *Tgfb2* expression was nearly four-fold lower (Figure 4F) while *Tgfb1* expression was also significantly lower but by a much smaller magnitude. At the transcript level, the directional changes of the PLR genes were the same as observed by proteomics, with *Mmp13*, *Ctsk*, and *Acp5* being significantly higher in old bone.

To better localize age-related TGF-beta signaling factors, we used RNA ISH to examine the expression of *Tgfb2*. We found that *Tgfb2* is predominantly expressed by osteocytes in young-adult and aged bone (Figure 4G). On the periosteal surface, less than 10% of periosteal surface cells expressed *Tgfb2*; this percentage significantly decreased with age (9% vs. 3%, *p* = 0.005, Figure 4H). On the other hand, over 50% of osteocytes in young-adult bone expressed *Tgfb2*, and this percentage also decreased in old bone (58% vs. 39%, *p* = 0.0005, Figure 4I). The areal density of *Tgfb2*-expressing osteocytes was approximately half in old bone (495 vs. 272 cells/mm^2^, *p* = 0.0005, Figure 4J). Therefore, both proteomic and transcriptomic approaches revealed baseline differences in TGF-beta signaling, particularly related to Tgfb2, which may be involved in PLR.

### The proteome differed more with age than mechanical loading

**Figure 5:**
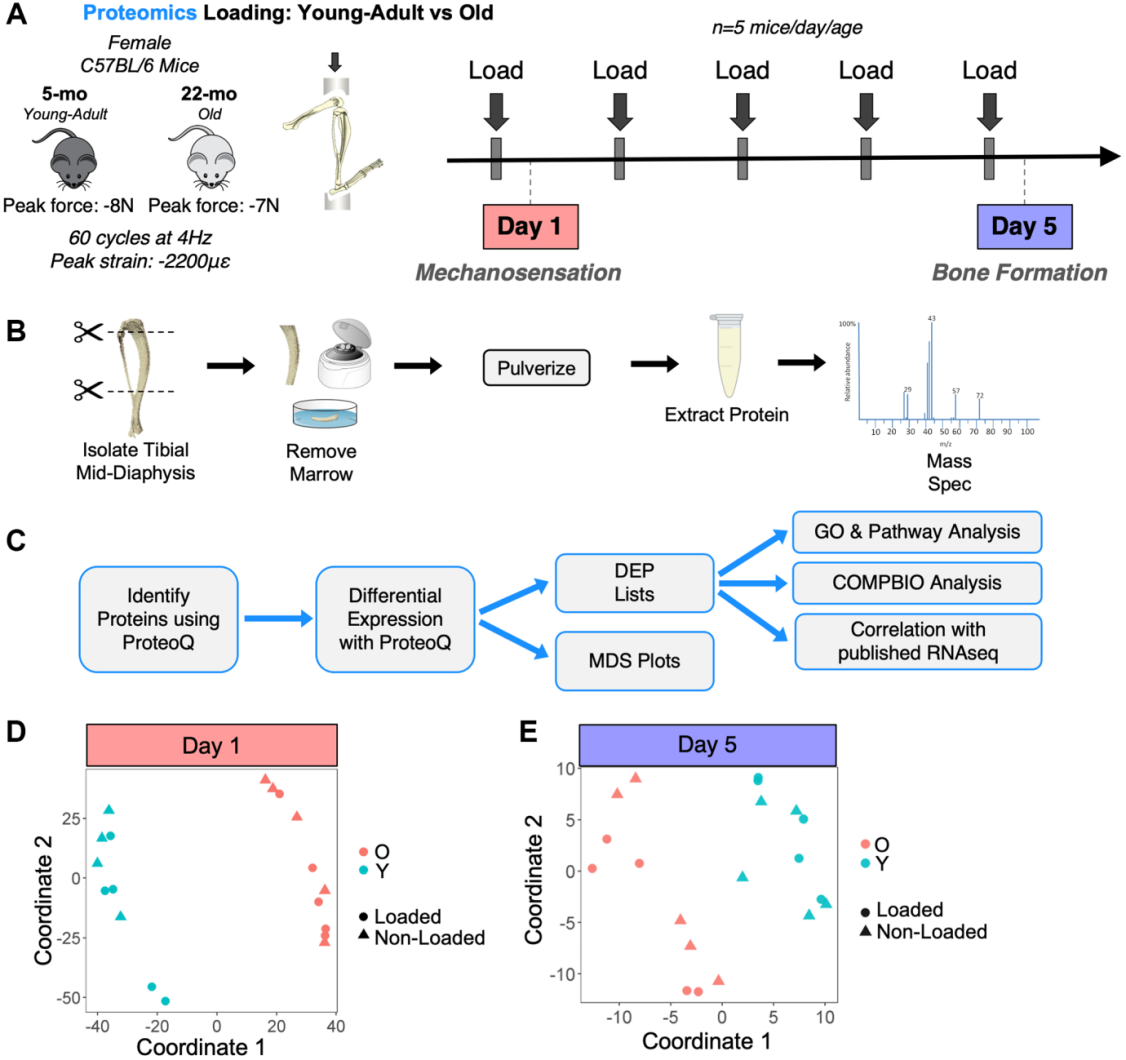
**Experimental design for proteomics loading experiment.** (**A**) 20 female C57BL/6N mice (5 mice/day/age) were subjected to daily *in vivo* axial tibial compression for either 1 (Day 1) or 5 (Day 5) bouts and sacrificed 6 hours after their final bout of loading. (**B**) The loaded (right) and non-loaded (left) tibial mid-diaphyses were isolated, removed of marrow, and snap frozen. Proteins were extracted in 4% SDS. Proteins for all 40 samples were then analyzed by proteomics using a tandem mass tag (TMT)-11 design. (**C**) Proteomics raw data were analyzed, and differential expression analysis was performed using ProteoQ. An unadjusted *p*-value cutoff of 0.05 and a fold-change threshold of 1.1 were used to identify loading-regulated proteins for downstream analyses, which included gene ontology (GO), PANTHER pathways, COMPBIO, and correlation with previously published RNA-seq data [11]. (**D**) At Day 1, multidimensional scaling (MDS) showed that the strongest differences were between ages rather than with loading status. (**E**) A Day 5, MDS showed slightly better separation between loaded and non-loaded samples, but the strongest separation was still between ages.

We next sought to study the proteomes of bones from young-adult and old mice following a mechanical loading stimulus. Tibias from a separate set of young-adult and old mice were loaded *in vivo* for 1 or 5 days (Figure 5A). Time points were selected to sample distinct phases of the loading response: early mechanosensation (day 1) and active bone formation (day 5). Paired loaded and non-loaded tibias were analyzed by proteomics (Figure 5B, 5C). After filtering, 2300 and 2140 proteins were detected by proteomics at days 1 and 5, respectively (Supplementary Figure 1). Multidimensional scaling (MDS) showed that young-adult samples separated from old samples regardless of loading status at day 1 (Figure 5D). As expected, at day 5, when bone formation is actively occurring [11], samples separated marginally better based on loading status, but the strongest proteomic differences were still due to age status (Figure 5E).

The top 10 upregulated and downregulated proteins following loading were compiled for each age and day combination (Table 2). Using the same DEP criterion of an FDR <0.05 to compare loaded and non-loaded samples, only five proteins (SMPD3, FKBP7, SSR3, EIF4G1, and EIF2S3X) reached significance across the entire loading experiment, all upregulated in young-adult bones at day 5. SMPD3, a sphingomyelinase that produces ceramide [90], was the most significantly upregulated protein. Ceramides are bioactive lipids that play a role in triggering proliferation and differentiation [91]. SMPD3 has GWAS associations with both BMD and height and is known to be important in bone for development [92] and fracture healing [93]. FKBP7 is a calcium-binding molecular chaperone. In old bones, neither protein was significantly upregulated following loading at day 5. Given that *Smdp3* and *Fkbp7* were also previously identified as upregulated DEGs (7- and 3-fold up, respectively) in young-adult mice but downregulated DEGs in old mice (3- and 2-fold down, respectively) [11], they may play a role in the diminished loading-induced bone-formation with aging. SSR3, a signal sequence receptor important for protein translocation across the ER membrane, was also significantly upregulated at the protein level. It was also a loading DEG [11] and has been linked to BMD and height, although little is known about its direct role in bone cells. The last upregulated DEPs were EIF4G1 and EIF2S3X, which both play a role in translation, and may reflect increased matrix synthesis during the loading-induced bone formation response. Notably, in young-adult mice at day 5, we also detected a trend toward reduced MMP13, consistent with our recent findings using gene microarray and IHC [94].

**Table 2:** Top 10 differentially expressed proteins (loaded vs. non-loaded) in young-adult and old mice following 1 or 5 days of loading, ranked by *p*-value.

**Young-adult**								
	**Day 1**				**Day 5**			
	**Protein**	**FC**	***p*Val**	**FDR**	**Protein**	**FC**	***p*Val**	**FDR**
*Upregulated*	LEMD2	1.5	1.7E-04	0.09	SMPD3^✱^	1.8	3.7E-06	0.01
	GTPBP10	2.3	8.1E-04	0.24	FKBP7^✱^	1.5	2.7E-05	0.03
	CD36	1.6	9.4E-04	0.24	SSR3^✱^	1.3	5.1E-05	0.03
	MCPT4	1.7	1.1E-03	0.25	EIF4G1	1.3	6.1E-05	0.03
	FMNL3	1.5	1.5E-03	0.27	EIF2S3X	1.3	8.5E-05	0.04
	RANGAP1	1.3	1.7E-03	0.27	HDLBP^●✱^	1.3	2.3E-04	0.06
	DNAJA2	1.3	1.8E-03	0.27	RPS17	1.4	2.7E-04	0.06
	NDUFB5	1.7	2.2E-03	0.27	VKORC1^✱^	1.4	2.9E-04	0.06
	TMEM65	1.3	2.4E-03	0.27	TMED3^✱^	1.4	3.0E-04	0.06
	NDUFA11	1.6	2.8E-03	0.27	RPL26	1.4	3.1E-04	0.06
*Downregulated*	PGM3^✱^	−1.5	5.7E-05	0.06	SPP1^●^	−1.4	2.2E-04	0.06
	CA3	−1.8	1.6E-04	0.09	HSPA2	−1.3	4.5E-04	0.07
	PSMA1	−1.2	5.5E-04	0.23	F9	−1.3	1.9E-03	0.12
	PPP1CA	−1.5	8.3E-04	0.24	HBA	−1.7	2.8E-03	0.17
	GARS	−1.3	1.8E-03	0.27	HBB-B1	−1.5	3.3E-03	0.17
	CD68	−1.3	2.7E-03	0.27	F2	−1.2	3.8E-03	0.18
	MDH1	−1.2	2.8E-03	0.27	CTSK^●^	−1.6	3.8E-03	0.18
	TUBB5	−1.3	2.9E-03	0.27	TGFB1	−1.2	3.9E-03	0.18
	OLA1	−1.2	2.9E-03	0.27	SERPINC1	−1.1	4.0E-03	0.18
	CTSH	−1.4	3.3E-03	0.27	MMP13	−1.3	4.7E-03	0.20
**Old**								
	**Day 1**				**Day 5**			
	**Protein**	**FC**	***p*Val**	**FDR**	**Protein**	**FC**	***p*Val**	**FDR**
*Upregulated*	PYGB	1.5	4.4E-03	0.96	LPAR1	1.4	8.2E-05	0.09
	BCL2L13	1.2	4.7E-03	0.96	STX4	1.3	2.1E-04	0.12
	YIPF5	1.4	5.6E-03	0.96	LOXL2^✱^	1.7	3.9E-04	0.12
	RCN1^✱^	1.2	6.8E-03	0.96	PLOD1	1.3	4.0E-04	0.12
	STOM	1.2	8.8E-03	0.96	SEC11A	1.3	4.3E-04	0.12
	NDUFB6	1.4	8.9E-03	0.96	EMP3	1.3	4.8E-04	0.12
	ACAN	1.6	9.4E-03	0.96	ATP2B1	1.2	5.7E-04	0.12
	PTRH2	1.3	1.2E-02	0.96	CFP	1.4	8.6E-04	0.16
	UBE2D2	1.1	1.4E-02	0.96	VIM	1.2	9.9E-04	0.16
	JUP	1.2	1.4E-02	0.96	SLC43A3	1.5	1.1E-03	0.16
*Downregulated*	YBX1	−1.3	3.2E-03	0.96	CA3	−1.5	2.6E-04	0.12
	SEPTIN9	−1.2	3.5E-03	0.96	PEA15	−1.2	5.3E-04	0.12
	NRP2	−1.3	7.2E-03	0.96	GPI	−1.3	2.2E-03	0.20
	LASP1	−1.2	1.1E-02	0.96	FABP3	−1.6	2.4E-03	0.20
	CAPRIN1	−1.2	1.2E-02	0.96	PGK1	−1.3	2.6E-03	0.20
	GNB4	−1.2	1.2E-02	0.96	MDH1	−1.2	3.5E-03	0.22
	MMP13	−1.2	1.3E-02	0.96	LDHA	−1.4	4.9E-03	0.26
	CDC37	−1.2	1.6E-02	0.96	TPI1	−1.3	6.4E-03	0.28
	ATP6AP1	−1.2	1.6E-02	0.96	PVALB	−2.0	8.4E-03	0.33
	PRDX1	−1.2	1.8E-02	0.96	PPP2R1A	−1.1	9.2E-03	0.33

FC: Linear fold-change (loaded/non-loaded). Abbreviation: FDR: False Discovery Rate. Bold: Met criterion for DEP. DEG by RNA-seq [11] in same age in same direction or different direction at Day 3 (^●^) or Day 5 (^✱^)

### Proteomes following loading showed distinct pathway, protein class, process enrichment

**Figure 6:**
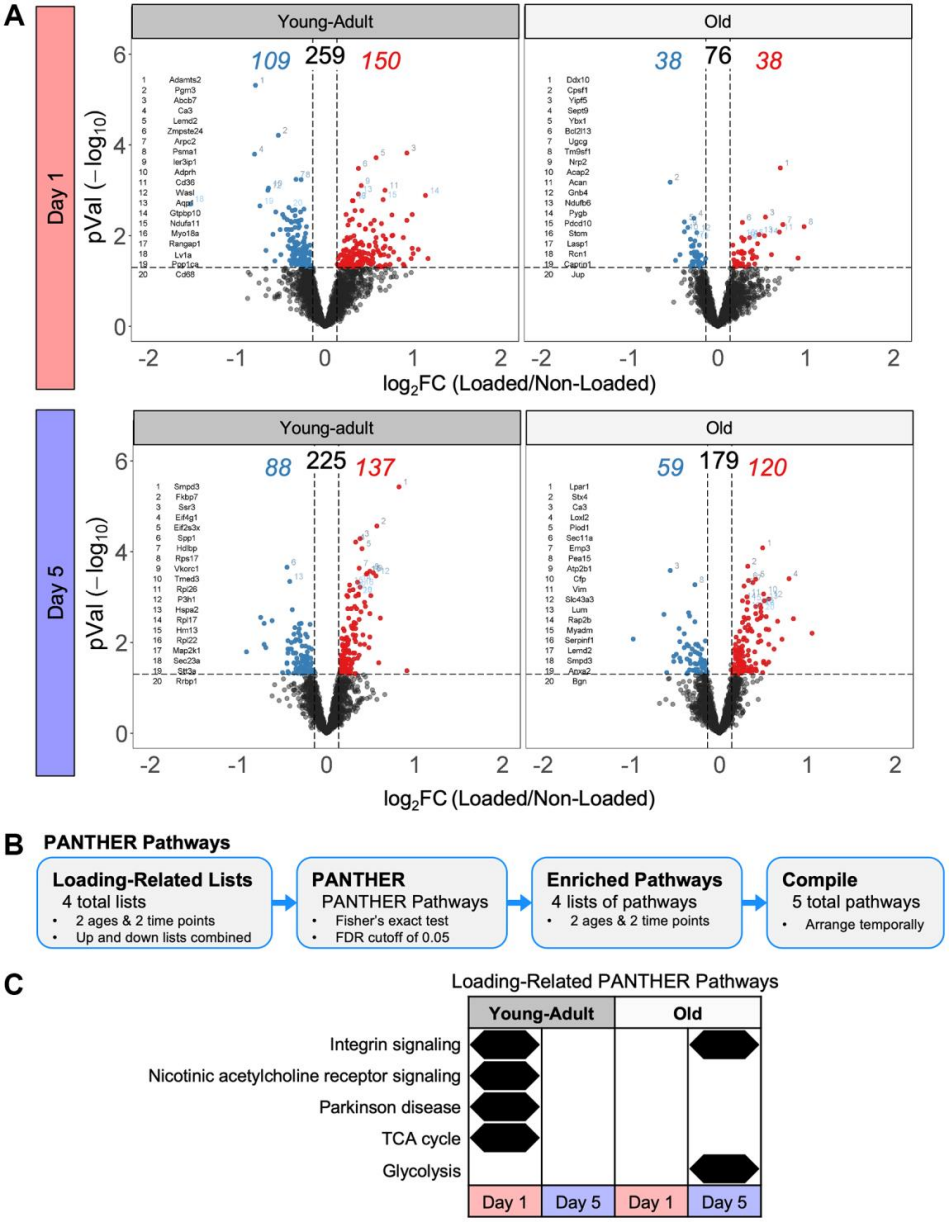
**Tibias from old mice had fewer protein-level changes following loading compared to young-adult mice.** (**A**) Volcano plots for each day and age combination following the loading experiment. The total number of loading regulated proteins is shown above with the upregulated number in red and the downregulated number in blue. (**B**) Loading-related proteins were input into PANTHER to identify enriched PANTHER pathways. (**C**) Loading-related PANTHER pathways were temporally arranged.

**Figure 7:**
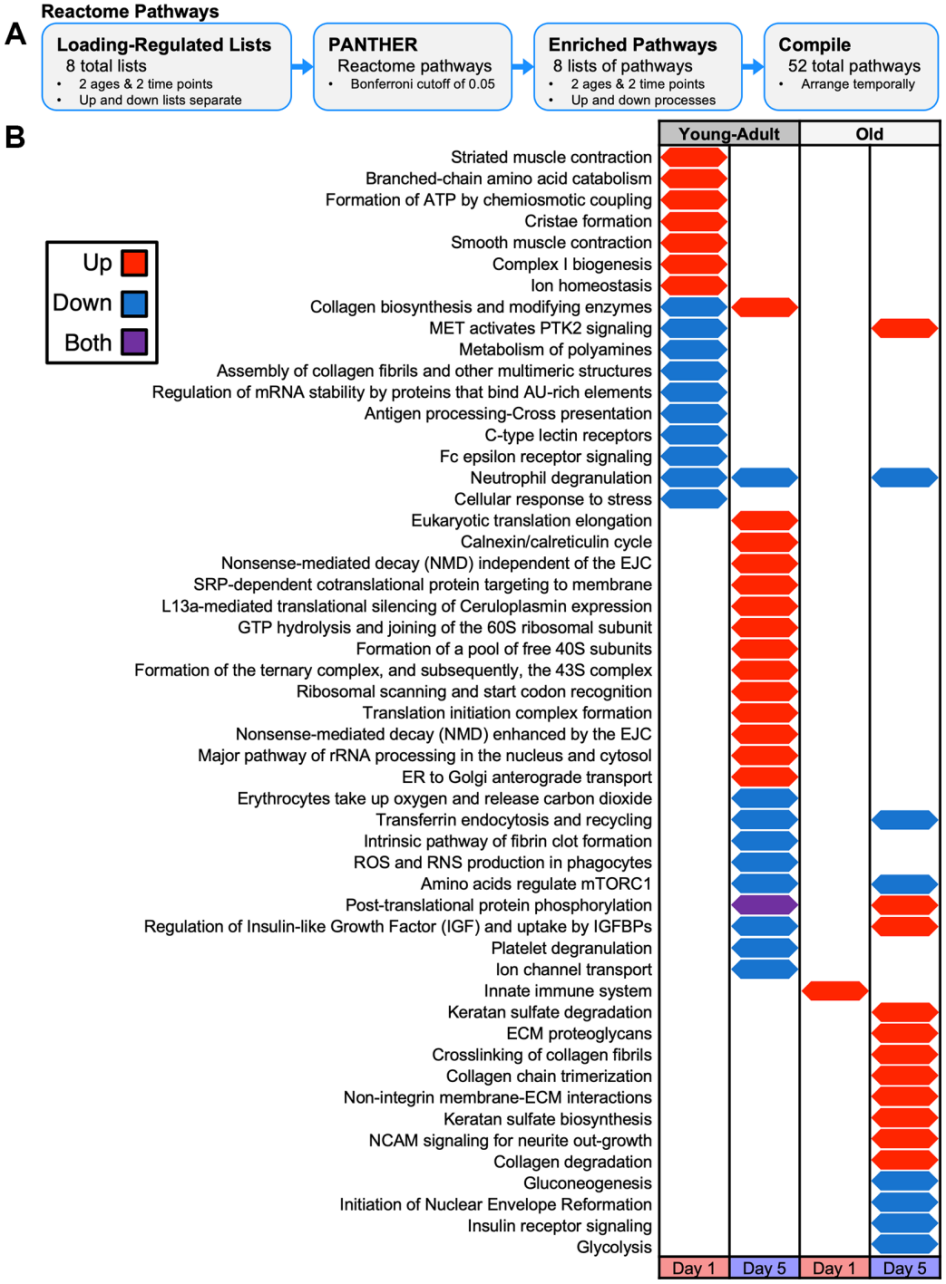
**Temporal map of PANTHER Reactome Pathways for proteomics loading experiment.** (**A**) Loading-responsive protein lists for each age and day combination (up and down lists separate) were input to PANTHER to identify enriched pathways. (**B**) Reactome pathways were arranged temporarily for upregulated or downregulated protein enrichment at each day and age combination. Both upregulated and downregulated lists were enriched for pathways.

As input for pathway analysis, we defined loading-responsive proteins using the following criteria: a linear fold-change of at least 1.1 (up or down) and an unadjusted *p*-value < 0.05. At day 1, tibias from young-adult mice had 150 upregulated and 109 downregulated loading-responsive proteins, whereas tibias from old mice had 38 upregulated and 38 downregulated loading-responsive proteins (Figure 6A). At day 5, tibias from young-adult mice had 88 upregulated and 137 downregulated loading-responsive proteins, whereas tibias from old mice had 59 upregulated and 120 downregulated loading-responsive proteins. We next examined the pathways that changed at the protein level following mechanical loading using combined upregulated and downregulated protein lists with PANTHER pathways (Figure 6B). We extended this pathway analysis with a more expansive and directional (upregulated and downregulated proteins separately) analysis: Reactome pathways (Figure 7A), gene ontology (GO) terms (Supplementary Figure 4), protein classes (Supplementary Figure 5), and COMPBIO themes (Supplementary Figure 6).

In young-adult bone at day 1, we observed enrichment of integrin signaling, nicotinic acetylcholine receptor signaling, Parkinson disease, and TCA cycle (Figure 6C). Consistent with the TCA cycle signal, Reactome pathway analysis indicated that upregulated proteins were enriched for pathways related to complex I and the formation of ATP by chemiosmotic coupling (Figure 7B). Protein class analysis showed enrichment of ATP synthase and dehydrogenase classes while GO processes related to mitochondrial electron transport. Actin-binding cytoskeletal proteins and ECM structural proteins showed protein class and GO enrichment, but interestingly, downregulated proteins were enriched for collagen-related processes. As expected at this early time point, we observed a prominent transcriptional signal.

In young-adult bone at day 5, no proteins were significantly enriched for any PANTHER pathway, but Reactome analysis showed that upregulated proteins were enriched for collagen biosynthesis. COMPBIO also identified an osteoblast theme within its top ten. The upregulated proteins were enriched for processes related to translation, consistent with the GO, protein class, and COMPBIO enrichments. Downregulated proteins at day 5 showed enrichment for amino acid regulation of mTORC1 and regulation of insulin-like growth factor.

In old bone at day 1, no proteins were significantly enriched for any PANTHER pathway, but Reactome analysis indicated an immune response. Protein class analysis indicated enrichment of calcium-binding proteins and ribosomal proteins while top COMPBIO themes related to ubiquitin degradation, actin filament dynamics, and RHO GTPase activity.

In old bone at day 5, we observed enrichment of integrin signaling and glycolysis. Reactome pathway analysis indicated that downregulated proteins were enriched for glycolysis and gluconeogenesis, consistent with the GO process enrichment and the protein class enrichment of isomerases and dehydrogenases. Downregulated proteins also showed enrichment for amino acid regulation of mTORC1, as in the young-adult samples. Upregulated proteins were enriched for collagen-related pathways, consistent with the GO and protein class enrichment related to the ECM. COMPBIO analysis reinforced these findings and also indicated changes in nuclear lamins/envelope. In contrast to the GO analysis in young-adult samples at this time point, we observed enrichment of processes related to transcription rather than translation, potentially suggesting a delay in the bone formation cascade with age.

## DISCUSSION

Our main objectives were (1) to compare the proteome and transcriptome of tibias from young-adult and old mice under baseline conditions, and (2) to define the changes in the bone proteome in response to mechanical loading. First, we successfully developed a proteomics method to detect protein-level changes in marrow-free cortical bone. Second, we employed this method to perform paired proteomics and RNA-seq on tibias of young-adult (5-month) and old (22-month) C57BL/6N female mice at baseline. The correlation between the proteome and transcriptome in bone was moderately positive (*ρ* = 0.40, *p* < 0.001), in line with other tissues [31, 32]. Differential expression analysis of young-adult vs. old bones at baseline identified 183 DEPs and 2290 DEGs. Eight of the shared differentially expressed targets had previously been identified as being important determinants of BMD through GWAS. We used RNA ISH to characterize the expression of Asrgl1 and Timp2, which have been understudied in bone. At baseline, old bones displayed diminished TGF-beta signaling and alterations in extracellular matrix (ECM) and matrix-metalloproteinases protein and transcript levels. We identified that *Tgfb2* was the most reduced *Tgfb* transcript in old bone and showed that *Tgfb2* was predominantly expressed by osteocytes. Third, we used proteomics to compare the loading responses between tibias of young-adult and old mice. Overall, we defined only modest loading-related changes in the proteome relative to the robust age-related differences. Nonetheless, we identified a handful of significant DEPs and were able to characterize the loading-responsive proteins and pathways in young-adult and aged mice following 1 and 5 days of loading. Specifically, compared to young-adult mice, old mice had fewer protein-level changes at both days and had enrichment of distinct biological pathways, such as those related to the nuclear envelope.

One previous study examined the proteome of exosomes isolated from bones of aged mice [16]. Another study characterized the loading response in bones from a small number of rats [18]; however, it is unclear whether these authors [18] removed the cellularly rich bone marrow for their analysis. Other whole-tissue studies of bone have been performed on relatively large tissue samples, namely canine skull [17], rat ulnas [18], mouse calvarias [19], and human femoral necks [20]. Here, we demonstrated the feasibility of a proteomic analysis of whole cortical tissue from mouse long bones, a widely used model system in skeletal biology.

We also report the first estimate of the correlation between the proteome and transcriptome in bone tissue, Spearman *ρ* = 0.40. Initial correlations for 12 non-bone tissues ranged from *ρ* = 0.41–0.55 [32]. More recently, a group used data from the GTEx project [95] and human proteome map [96] to correlate the transcriptome and proteome in 14 non-bone tissues, finding a range from *ρ* = 0.36–0.5 [31]. Our value of *ρ* = 0.40 for bone is in line with these estimates for other tissues. We suspect that this relatively low value has both a biological and technical basis. First, differences between RNA and protein expression may result from the post-transcriptional and post-translational regulation of many proteins, as has been demonstrated in bone [97]. It may also reflect a temporal lag between when a gene is transcribed and when the corresponding protein is expressed, and likewise may reflect the abundance of extracellular matrix proteins in bone that persist long after transcription has stopped. We note that our correlation estimate is for bone at a baseline state, and it is possible that evaluating the correlation following an anabolic stimulus such as mechanical loading or pharmacological treatment may provide a different result. Finally, technical differences between the sampling depth of sequencing versus mass spectrometry methods may have also contributed to our result. Nonetheless, our finding reinforces the idea that the transcriptome falls short of capturing the full biological picture in most model systems and shows that bone is no exception [98, 99].

One of the nine hallmarks of aging—loss of proteostasis [100]—has been relatively understudied in the bone outside of the context of autophagy models [101, 102]. Proteomics is particularly well suited for this application given its ability to detect both low-magnitude transcriptional changes that accumulate over time and changes in protein that occur independent of transcriptional control (e.g., due to chaperone or proteosome dysfunction). At the protein level, but not at the RNA level, we identified age-related differences in ER-associated decay (ERAD), ubiquitination, and chaperone proteins. It’s been hypothesized that chaperones in osteocytes are important for their ability to adapt and live for years while deeply embedded in mineralized matrix [103], perhaps through autophagy [104]. Thus, these changes may partly underlie the reported age-related death of osteocytes and degeneration of the osteocyte lacunocanalicular network [105]. Of note, osteocyte apoptosis was reported to be reduced by mechanical loading [106]. Given the finding that voluntary treadmill running can partly restore the muscle proteome to its youthful state [107], exercise-induced bone loading or a more longitudinal loading regimen may hold potential to rescue the age-related changes in the bone proteome.

In aged bone, we found prominent alteration of lamins and cytoskeletal elements at the protein level. Previous *in vitro* work has shown that prominent nuclear/lamin and actin cytoskeletal features can distinguish mature osteocytes from the earlier osteoblast lineage [103]. Specifically, lamins are thought to play a role in maintaining healthy bone by maintaining differentiation and survival [108], and lamins and actin cytoskeletal elements are thought to be important for mechanosensation in a variety of tissues [109]. Therefore, these alterations in lamins/envelope and cytoskeletal elements may have a mechanistic connection to the diminished loading response with aging.

Our multi-omics approach identified the targets Asrgl1 and Timp2, proteins that—despite showing lower expression with age and being associated with BMD [39]—remain understudied in bone. We used RNA ISH to validate our findings and characterize their expression patterns in young-adult and old bone. The function of *Asrgl1*, which has asparaginase but not glutaminase activity, remains unclear. We learned that in young-adult bone, approximately half of osteocytes express *Asrgl1 (*asparaginase and isoaspartyl peptidase 1), while only a quarter of the periosteal surface is covered by *Asrgl1-*expressing cells. In old bone, this percentage of *Asrgl1*-expressing surface cells is halved, consistent with the lower abundance of Asrgl1 in old bones. Timp2 (Tissue inhibitor of metalloproteinases 2) is known to inhibit MMP function, especially in calvariae [110], where MMPs are known to play a role in osteoclastic resorption [111]. Here, we learned that in normally developed long bones, *Timp2* is expressed by most osteocytes, and most of the periosteal surface is covered by *Timp2*-positive cells. In old bone, the total surface cell expression of *Timp2* is halved and osteocyte expression also decreases.

At baseline, old mice displayed diminished TGF-beta signaling and showed alterations in MMPs/ECM compared to young-adult mice. Our finding that *Tgfb2* is predominantly expressed by osteocytes is consistent with a previous developmental study that showed that *Tgfb2* expression was localized in osteocytes but not the periosteum or marrow [112]. While *Tgfb1* and *Tgfb2* were expressed at comparable levels in young-adult bones, *Tgfb2* was the most reduced *Tgfb* transcript type with age. Although *Tgfb1* is frequently described as the predominant TGF-beta isoform in bone, *Tgfb2* may play an underappreciated role in bone homeostasis and aging; it is already known to be important in the development of both the axial and appendicular skeletons [113]. Additionally, the lack of phenotypic overlap between Tgfb2 KO, Tgfb1 KO, and Tgfb3 KO models suggests independent, non-compensatory functions of the isoforms [113]. Our results seem to differ from a report that TGFB1 increases with aging [114]. We speculate that this discrepancy may be due to differences in the samples; we analyzed diaphyseal cortical bone, whereas Li et al. analyzed the metaphysis, which contains more trabecular bone [114] and may be enriched for different progenitors. Thus, in different regions of bone, there may be distinct TGF-betas isoforms and how they change with aging may be unique.

Based on previous loss-of-function models of TGF-beta signaling in osteocytes [89], we had expected to find reduced MMP expression in old bone. Nonetheless, despite the lower TGF-beta signaling in aged bone, MMP13 was also more highly expressed. Given that osteocytes comprise ~95% of bone cells and that osteocyte-derived MMP13 has been shown to be critical for maintaining PLR, we attribute this MMP13 expression to osteocytes [52, 53] but efforts to histologically localize MMP13 at the protein level in these samples were not reproducible. Overall, we speculate that the alterations in TGF-beta, Timp2, and MMPs relate to dysregulated PLR with aging.

We designed the loading experiment to extend our understanding of the loading response at the protein level, which has not been addressed by prior *in vivo* -omic study designs. The loading response of bone is known to be partly regulated post-transcriptionally [115]. In fact, following loading, sclerostin is actively degraded by lysosomes [97]. Previously, however, most proteomic studies into the mechanoresponsiveness of bone have been *in vitro* [103, 15]. Given that the mechanosensing osteocyte lacunocanalicular network degenerates with aging [105], we hypothesized that we would detect, protein-level differences at an early time point following loading. Therefore, we assessed the response at day 1, before bone formation began (during mechanosensation). We also expected to detect differences between ages during bone formation, so we assessed the response at day 5, when bone formation is actively occurring. At the protein-level, the loading-related changes were modest compared to the age-related differences. As in our previous transcriptomic results, old bone had fewer protein-level changes at both days compared to young-adult bone. Transcription signal was observed at day 1 in young-adult bone but only at day 5 in old. By day 5, young-adult bone showed a translation signal, so this may represent a delay in the aged loading-induced bone formation cascade. Additionally, at day 5, only old bone had downregulation of pathways related nuclear lamins/envelope, which may play a role in the blunted bone formation response.

Overall, a proteomic analysis of bone at the whole-tissue level presented two major challenges: (1) the large pre-existing pool of extracellular proteins in bone, and (2) the multiple cell types contained within marrow-free bone. First, we found that the loading-related changes at the protein level were relatively modest. We hypothesize that this may be attributable to the large pre-existing pool of proteins contained within matrix-rich cortical bone, which could mask acute, stimulus-induced changes. We suggest that future efforts to study proteomic changes in bone following acute interventions employ strategies to label and enrich for newly translated proteins. In the present study, we did not enrich for post-translational modifications (i.e., phosphorylation or acetylation), which are thought to mediate many mechanosensitive pathways [116] and have been demonstrated to acutely change during exercise [117]. We hypothesize that adding such an enrichment step would enable a more sensitive assessment of protein level changes following loading. Second, we studied bulk cortical bone, which despite taking extreme care to remove all marrow and adherent muscle, still contains all major bone cell types (i.e., osteocytes, osteoblasts, osteoclasts, and bone lining cells) at a variety of differentiation stages in addition to investing structures like nerves and vessels. Due to this mixture of cell types and states, important changes in any one cell type may have been ‘averaged out’ and missed in this study [99]. In addition, with our present bulk design, it is impossible to attribute expression of a certain factor to a particular cell type or lineage. In the present study, we addressed this limitation by employing complementary histological validation efforts to localize key targets. However, histological validation is inherently low-throughput and does not lend itself to systems biology approaches for studying bone. Future efforts may benefit from employing cell-specific markers for enrichment, such as using a Cre driver with the bio-orthogonal non-canonical amino acid tagging (BONCAT) platform [118].

Our study had several other limitations. First, as in other studies, the higher variability in aged samples was a challenge for detecting experimental differences. Second, the TMT-11 study design to facilitate optimal peptide quantification limited our sample number to 5 per group and is known to cause ratio compression, which likely underestimated our fold-changes [119]. As label-free quantification becomes more common, such an approach should be considered in future proteomics studies on bone tissue [120]. Third, despite detecting many intracellular proteins (e.g., we detected most of the proteins involved in glycolysis), our proteomic sampling depth was relatively low for studying intracellular changes. Given that numerous proteins of interest in bone (e.g., Wnt ligands or transcription factors) are lowly expressed relative to matrix proteins, combined targeted and multiplexed strategies may aide in investigating such proteins [121]. Fourth, while we validated several of our omics findings with *in situ* hybridization, as with other big data studies, our interpretations remain speculative without functional validation. While organ culture models hold potential for delving into some of these mechanisms, functional validation was outside of the scope of the current study. Future efforts using *in vitro* and *in vivo* approaches are required to further understand the mechanistic role of the targets we identified.

In summary, we developed a proteomics method to detect protein-level changes in cortical bone with aging. Based on paired proteomics and RNA-seq on tibias of young-adult and old mice at baseline, we reported the first estimate of the correlation between the proteome and transcriptome in bone (*ρ* = 0.40, *p* < 0.001). We found many differences with aging at both the protein- and RNA-level, and characterized the expression of two targets (Asrgl1, Timp2), which may be important for skeletal aging. In addition, old bones displayed diminished TGF-beta signaling at baseline, and osteocyte-derived *Tgfb2* was the most reduced *Tgfb* transcript. Proteomics detected substantial age-related differences in proteostasis related to ERAD, chaperone proteins, and ubiquitination. Finally, we used proteomics to compare the loading responses between tibias of young-adult and old mice. We found that the proteome differed more with age than loading status and identified loading-induced protein-level changes in both ages, including enrichment of distinct pathways in aged bone. We conclude that proteomics is a promising approach to study bone biology and detect protein-specific changes in aging.

## MATERIALS AND METHODS

### Mice

Female C57BL/6N mice were obtained at 5 months (young-adult) and 22 months (old) from the aged rodent colony at the National Institute on Aging, which is managed by Charles River Laboratories (Figure 1A). Female mice were selected because: (1) osteoporosis is more prevalent in females [1]; (2) male mice often fight, which can impact the skeleton and confound the effects of loading [122]; and (3) to facilitate comparison with our previous RNA-seq study, which used female mice [26–30, 11]. Across all experiments, 58 mice (34 young-adult and 24 old) were used in this study. Mice were housed in groups of up to five animals of the same age and kept on a 12-hour light/dark cycle under standard conditions with *ad libitum* access to water and chow (Purina 5053 and 5058). All animal work was approved by and in compliance with the Washington University IACUC. All included mice were healthy throughout the experiment. For the baseline experiment, we performed paired proteomics and RNA-seq on tibias of young-adult (5-month) and old (22-month) C57BL/6N female mice not subjected to any interventions (Figure 1A). For the loading experiment, we used proteomics to compare the loading responses between tibias of young-adult and old mice following 1 or 5 days of axial loading (Figure 5A). MicroCT of the right femurs from the mice used in the baseline comparison confirmed the expected age-related changes in bone, specifically decreases in distal cortical bone area, distal cortical thickness, trabecular bone volume per total volume (BV/TV), and trabecular number (Figure 1B–1G and Supplementary Figure 7).

### *In vivo* mechanical loading

In the loading experiment, mice were loaded for either 1 or 5 bouts of daily loading. Mice were anesthetized (3% isofluorane) and subjected to loading each morning for the specified number of days. With the mouse prone, the right leg (tibia) was placed vertically in the loading fixture with the knee positioned superiorly in a semi-spherical cup (10 mm diameter) attached to the system actuator, and the foot held in a static fixture inferiorly (20° of dorsiflexion). A preload (−0.5 N) was applied, and tibias were subjected to axial compression for 60 cycles/day (4Hz haversine waveform) using the Electropulse 1000 materials testing system (Instron). This loading protocol is anabolic for both cortical and trabecular bone in young-adult mice [123]. We used a strain-matched study design. Based on prior strain gauging analyses [124] and consistent with our RNA-seq study, age-specific peak forces of -8N and -7N were selected for the 5- and 22-month-old mice, respectively, to engender average peak compressive periosteal strains of −2200 µε at the cortical mid-shaft [28]. Corresponding tensile strains on the anterio-medial surface were approximately 1200 µε [124]. After each loading bout, buprenorphine (0.1 mg/kg subcutaneously) was given to mitigate pain from loading [125], and mice were returned to their cages to resume unrestricted activity. The left tibias served as non-loaded, contralateral controls. Six hours after their final loading bout, mice were euthanized by CO_2_ asphyxiation.

### Tibial isolation

For proteomics and RNA-seq analyses, right and left tibias were stripped of muscle, cut at the distal tibiofibular junction and 2 mm distal to the tibial plateau, placed in ice-cold PBS, centrifuged to remove the bone marrow [125, 126], flushed with PBS, and snap frozen in liquid nitrogen. Samples were stored at -80°C until protein or RNA extraction. For the histology assays, the left tibias were cut at the ankle and above the tibial plateau and trimmed of muscle with care not to disturb the periosteum; samples were immediately fixed in 10% neutral-buffered formalin (NBF).

### Protein extraction by homogenization and sonication

Preliminary experiments were performed to compare the protein yield and peptide detection of several tissue homogenization methods (Supplementary Figure 8). Frozen tibial samples were homogenized with the cryoPREP (Covaris CP02) pulverizer in small impactor bags (tissueTUBES, Covaris # 520071), as previously described in other tissues [127]. With the bag partly submerged in liquid nitrogen, the bone was transferred into the bag, and the cap was lightly screwed on (to allow air to escape during the impact). The CryoPrep was activated on impact level 5. After impact, 100 µL of SDS (4 % SDS, 100 mM Tris-HCl, pH 8.0) at room temperature (RT) were added to the bag. A pipette was used to spread and agitate the buffer within the bag to cover all bone chips. Eventually, the bone lysate became ‘stringy’ and was pushed into a corner of the bag. Using a pipette (with cut tip), the lysate was transferred to a sonication vial (Covaris #520130). After incubating at RT for 5 min, the samples were sonicated in the S220X focused-ultrasonicator (Covaris) for 4 min with the following settings: PIP: 500 Watt, cycles per burst: 500, duty factor: 10%. Sonicated samples were transferred to 1.7 mL Eppendorf tubes and spun at 14,000 × g for 15min at 8°C to pellet debris. The supernatant was collected, transferred to a new 1.7 mL tube, and 2 µL were reserved for protein concentration determination, according to BCA kit instructions (Pierce #23227); the remaining lysate was reduced with 100 mM DTT followed by heating at 95°C for 10 min. Samples were stored at −80°C.

### Peptide preparation

The samples were digested using a modification of the filter-aided sample preparation (FASP) method, as previously described [128]. The reduced samples were mixed with 200 µL of 100 mM Tris-HCL buffer, pH 8.5 containing 8M urea (UA buffer) and transferred onto the top chamber of a 30,000 MWCO cutoff filtration unit (Millipore #MRCF0R030) and spun in a microcentrifuge at 14,000 × g for 10 min. An additional 200 µL of UA buffer was added to the top chamber of the filter unit and the filter was centrifuged at 14,000 × g for 15–20 min. The flow through was discarded, and the proteins were alkylated by adding 100 µL of 50 mM Iodoacetamide (Pierce #A39271) in UA buffer to the top chamber of the filtration unit and gyrating at 550 rpm in the dark at RT for 30min using a thermomixer (Eppendorf #05-400-205). The filter was spun at 14,000 × g for 15 min, and the flow through was discarded. Unreacted iodoacetamide was washed through the filter with two sequential additions of 200 µL of UA buffer and centrifugation at 14,000 × g for 15–20 min after each addition. The urea buffer was exchanged into digestion buffer (DB) consisting of 50 mM ammonium bicarbonate buffer at pH 8. Two sequential additions of 200 uL of DB with centrifugation after each addition to the top chamber were performed. The top filter units were transferred to a new collection tube, and 1 micro unit of LysC (Wako Chemicals #129-02541) was added. Samples were digested at 37°C for 2hr. After LysC digestion, 1 µg of sequencing-grade trypsin (Promega #V5113) was added, and samples were digested overnight at 37°C. The filters were spun at 14,000 × g for 15min to collect the peptides in the flow through. The filter was washed with 50 µL of 100 mM ammonium bicarbonate buffer, and the wash was collected with the peptides. In preparation for desalting, peptides were washed 3 times in 1mL ethyl acetate followed by acidification to 1% (vol/vol) trifluoroacetic acid (TFA) final concentration. The peptides were desalted using porous graphite carbon two micro-tips (Glygen BIOMEK NT3CAR) on a Beckman robot (Biomek NX), as previously described [129]. The peptides were eluted with 60% (vol/vol) acetonitrile in 0.1% TFA (vol/vol) and dried in a SpeedVac (Thermo Scientific Savant DNA 120) after adding TFA to 5% (vol/vol). The peptides were dissolved in 20 µL of 1% (vol/vol) acetonitrile in water. An aliquot (10%) was removed for quantification using the Pierce Quantitative Fluorometric Peptide Assay kit (Thermo Scientific, Cat. No. 23290). The remainder of the enriched samples and reference pool sample were transferred into a 0.5 mL Eppendorf tube, dried in the Speed-Vac and dissolved in 12 µL of HEPES buffer (100 mM, pH 8.0) (Sigma, H3537).

The peptides were labeled with tandem mass tag (Thermo Scientific TMT10 or TMT11-131C) reagents according to the manufacturer protocol. For the baseline comparison between old and young-adult bones, the samples were analyzed in a single plex using TMT-10 (Supplementary Figure 9). For the loading comparison at each time point (Figure 5A), young-adult and old samples (paired tibias from *n* = 5 mice per age) were analyzed on two age-specific runs separately. An 11th bridge sample containing pooled young-adult and old samples per time point was created to facilitate quantitative comparisons between ages. The labeled samples that were assigned to each ten- or eleven-plex were pooled, dried, and dissolved in 120 µL of 1% FA. The TMT-labeled samples were desalted as described above for the unlabeled peptides. The eluates were transferred to autosampler vials (Sun-Sri #200046), dried, and stored at −80ºC.

### Proteomics nano-Liquid Chromatography–Mass Spectrometry (nano-LC-MS)

The samples in formic acid (1%) were loaded (2.5 µL) onto a 75 µXm i.d. × 50cm Acclaim PepMap 100 C18 RSLC column (Thermo Fisher Scientific) on an EASY *nano*LC (Thermo Fisher Scientific) at a constant pressure of 700bar at 100% A (0.1%FA). Prior to sample loading, the column was equilibrated to 100% A for a total of 11 µL at 700bar. Peptide chromatography was initiated with mobile phase A (1% FA) containing 5% B (100%ACN, 1%FA) for 1 min, then increased to 15% B over 108 min, to 25% B over 87 min, to 35% B over 40 min, to 70% B in 6 min, to 95% B over 2 min and held at 95% B for 18 min, with a flow rate of 300 nL/min. The data were acquired in data-dependent acquisition (DDA) mode. The full-scan mass spectra were acquired with the Orbitrap mass analyzer with a scan range of *m/z* = 375 to 1500 and a mass resolving power set to 70,000. Twelve data-dependent high-energy collisional dissociations were performed with a mass resolving power set to 35,000, a fixed lower value of *m/z* 100, an isolation width of 1.2Da, and a normalized collision energy setting of 32. The maximum injection time was 60ms for parent-ion analysis and 120ms for product-ion analysis. The target ions that were selected for MS/MS were dynamically excluded for 20sec. The automatic gain control (AGC) was set at a target value of 3e6 ions for full MS scans and 1e5 ions for MS2. Peptide ions with charge states of 1 or ≥7 were excluded for HCD acquisition.

### Proteomics analysis

PSM files were imported into ProteoQ (https://github.com/qzhang503/proteoQ) for normalization, quantitation, and analysis. Data were further explored using principal component analysis and multidimensional scaling. At baseline, differential expression analysis was performed between young-adult and old samples. For the loading comparison, differential expression analysis was performed separately within an age for each time-point between loaded and non-loaded samples. Linear modeling was performed using the contrast fit approach in *limma* [130] to assess the statistical significance in protein abundance differences between indicated groups of contrasts. To facilitate the high-confidence assignment of proteins, we used a strict criterion of peptide spectral matches (PSM) ≥3 to call each protein.

### RNA isolation and library prep

Frozen bones were homogenized using a mikro-dismembrator (Braun Biotech International), and total RNA was extracted using TRIzol (Ambion) with the RNeasy Kit (Qiagen # 74004). Total RNA integrity was determined using the 4200 Tapestation, and concentrations were measured with the Qubit fluorometer. The median RNA integrity number (RIN) was 5.8 with a range of 5.1–6.8 (Supplementary Figure 1). No samples were excluded. Library preparation was performed with 500 ng of total RNA, and ribosomal RNA was blocked using FastSelect reagents (Qiagen) during cDNA synthesis. RNA was fragmented in reverse transcriptase buffer with FastSelect reagent, and mRNA was reverse transcribed to yield cDNA using SuperScript III RT enzyme (Life Technologies) and random hexamers.

### RNA sequencing and aligning

A total of 14 samples were subjected to RNA-seq (Figure 1B) by the Washington University Genome Technology Access Center. Samples were indexed, pooled, and sequenced on an Illumina NovaSeq 6000. Basecalls and demultiplexing were performed with bcl2fastq2 (Illumina), and the reads were aligned to Ensembl release 76 using STAR (2.5.1a) [131]. Gene counts were derived from uniquely aligned, unambiguous reads using Subread:featureCount (1.4.6-p5) [132].

### RNA-seq analysis

To adjust for differences in library size between samples, normalization factors from gene counts were calculated using EdgeR [133]. Ribosomal genes and genes not expressed >1 count-per-million (CPM) in at least six samples were excluded from initial analyses. The size factors and matrix of counts were then imported into *limma* [130], and weighted likelihoods based on the observed mean-variance relationship were calculated using *voomWithQualityWeights* [134]. For the downstream analysis, genes that were not expressed (CPM = 0) in any samples were filtered out. Statistical model fitting and robustness were then assessed with multidimensional scaling (Supplementary Figure 1). Next, differential expression analysis between young-adult and old samples was performed using *limma*’s moderated *t*-tests, and the results were filtered for genes with Benjamini-Hochberg adjusted *p*-values < 0.05.

### Co-expression network analysis for module construction

For the RNA-seq data, weighted gene co-expression network analysis (WGCNA) was used to generate co-expression networks for genes that differed between young-adult and old samples [135]. The *Limma voomWithQualityWeights-moderated log2 counts-per-million* generated for the previous differential expression analysis were used as inputs. All genes were correlated across each other using Pearson correlations and clustered by expression similarity into unsigned modules using a power threshold empirically determined from the data. To identify modules associated with age, the eigengene of each module was determined and correlated with age status (young-adult vs. old).

### Gene ontology, pathway, protein class, and COMPBIO analyses

For the gene ontology (GO), pathway, and protein class analyses, the differentially expressed genes (DEG), differentially expressed proteins (DEP), or loading-regulated protein lists were input into PANTHER (version 14.1) [136] to identify enrichment. GO Slim was used for the GO analysis, and both PANTHER and Reactome pathways were analyzed in the pathway analysis. GO terms, pathways, and protein classes were arranged temporally by age and then alphabetically. For the COMPBIO (COmprehensive Multi-omics Platform for Biological InterpretatiOn, https://becker.wustl.edu/resources/software/compbio/) analysis, the described gene lists were input with the default parameters to determine the most prominent themes. The COMPBIO platform uses an ontology-free approach to generate a comprehensive and contextual map of the core biological concepts and themes associated with input entities. Specifically, it assembles the maps using contextual language processing algorithms to scan all PubMed abstracts to identify enriched concepts associated with the input entities. The platform utilizes conditional probability analysis to compute the statistical enrichment of biological concepts (processes/pathways) over those that occur by random sampling. Related concepts built from the input list of differentially expressed genes are clustered into themes (e.g., biological pathways/processes, cell types and structures) and further interconnected into groups.

### Bone fixation for histology and RNAscope *in situ* hybridization

After 32hrs of fixation in NBF at RT, tibias were washed in PBS and decalcified in 14% ethylenediaminetetraacetic acid (EDTA) for 18 days at 4°C. EDTA was changed daily for the first 3 days and then every other day thereafter. Full decalcification was confirmed with X-ray. The tibias were processed for transverse (cut at the proximal diaphysis) paraffin sectioning (5 µm) by the Musculoskeletal Research Center Histology Core.

### RNA *in situ* hybridization (ISH) using RNAscope

The manufacturer’s protocol for the RNAscope^®^ 2.5 HD Detection Reagents (ACD #322310) was followed except where noted. Paraffin sections were baked overnight at 60°C and deparaffinized. Hydrogen peroxide (ACD #322335) was applied for 10 min to quench endogenous peroxides, and pepsin (Sigma #R2283) was applied for pre-treatment at 37°C for 45 min. The remaining protocol was followed for the target probe hybridization (Supplementary Table 1), amplification, DAB reaction, and counterstaining. A negative and positive control probe were included with each batch, and for each round, the control results were as expected (Supplementary Figure 10). Slides were cover slipped with VectaMount (Vector #H-5000-60).

### Immunohistochemistry (IHC)

For MMP13, sections were deparaffinized and rehydrated. Antigen retrieval was accomplished using UNI-TRIEVE (Innovex Biosciences #NB325) at 60°C (water bath) for 30min. For the remaining staining, the Universal Animal IHC Kit (Innovex #329ANK) was used with slight modifications. Endogenous peroxidases were blocked with PEROX-BLOCK for 15 min at RT, and background staining was blocked using Fc-Block for 45 min followed by Background Buster for 45 min. Primary anti-MMP13 antibody (1:50 dilution in PBS, Abcam #ab39012) or normal rabbit IgG (Cell Signaling Technologies #2729) was incubated overnight at 4°C in a humidity chamber. The Multivalent Secondary Antibody was incubated for 10 min, and next, the Peroxidase (HRP) was incubated for 10 min. Finally, the DAB substrate was applied for 5 min, and enhanced with DAB enhance for 3 min. Sections were counterstained with hematoxylin (Vector #H-3404) and cover slipped using Advantage Mounting Medium (Innovex NB300). For SOST, the VectaStain ABC Goat IgG Kit (Vector Labs #PK-40005) and ImmPACT DAB (Vector #SK-4105) kits were used. Sections were deparaffinized and rehydrated, and no antigen retrieval was performed. Endogenous peroxidases were blocked with BLOX-ALL for 10 min, and background staining was blocked using 3% BSA with 1 drop of Normal Rabbit serum for 1hr. Primary anti-SOST antibody was prepared in the same blocking buffer (1:200) and incubated overnight at 4°C in a humidity chamber. After washing, the rabbit anti-goat secondary antibody was incubated for 30 min, followed by the ABC Elite reagent for 30 min, and finally the DAB reaction for 4min. Slides were counterstained with hematoxylin (Vector Labs #H-3404), dehydrated, cleared with Xylenes, and cover slipped with VectaMount (Vector #H-5000-60).

### Histological analysis and cell counting

Slides were imaged on a NanoZoomer 2.0-HT system (Hamamatsu) in brightfield at 40X. Images were blinded and analyzed with either BIOQUANT (OSTEO II) or ImageJ (Fiji) to quantify positive cells staining on the periosteal surface and intracortically (osteocytes). For the surface percent positive analysis, BIOQUANT was used to analyze the entire bone perimeter. If any part of the cell was positive or displayed puncta, that cell length was traced as positive. For the total surface expression analysis, ImageJ was used to manually isolate the periosteum, and the default color deconvolution (H-DAB) was used to isolate the periosteal DAB signal. Finally, after converting the DAB channel image to binary, the pixels were counted, converted to an area, and normalized per surface length. For the osteocyte analysis, in BIOQUANT, a 600 µm × 600 µm region of interest (ROI) was defined on the tibial apex, and positive and negative osteocytes (OCY) within the ROI were counted. The bone area within the ROI was also measured, and using these measures, we calculated both the percent positive (*posOCY*/*totOCY*) and areal density (*posOCY*/*ROI Area*) measures. On the surface, only the periosteum was analyzed because the marrow often separated from the endosteal surface, preventing reliable analysis of this surface.

### Statistical analysis

For both proteomics and RNA-seq in the baseline experiment, we defined DEGs and DEPs as targets that had a Benjamani-Hochberg adjusted *p*-value < 0.05. For the loading experiments, we compared loaded versus non-loaded samples within each age, and very few proteins reached the FDR <0.05. We performed a sensitivity analysis with a variety of parameters, and regardless of the criteria used, the overall trends in the number of loading-responsive proteins were consistent for each age and day combination (Supplementary Figure 11). Therefore, to facilitate downstream analysis on the most differentially expressed protein targets with loading, we relaxed to the following criteria: a linear fold-change cutoff of >1.1 (up or down) and an unadjusted *p*-value < 0.05. For the pathway analyses, a Fisher’s exact test was used with an FDR of 0.05. For the co-expression network analysis, the significance threshold was set using a Bonferroni correction (*p* = 0.05/44 = 0.0011). For the RNA ISH and other histological analyses, after observing consistent staining with the probes, we analyzed one slide per target (*n* = 7 per age). If the periosteum lifted from the slide, that slide was excluded from the periosteal surface analysis, resulting in *n* = 6 per age for some targets. An unpaired *t*-test was used to compare measures between young-adult and old bones (GraphPad Prism 9.0).

## Supplementary Materials

Supplementary Figures

Supplementary Table 1

## References

[1] Melton LJ 3rd, Chrischilles EA, Cooper C, Lane AW, Riggs BL. Perspective. How many women have osteoporosis? J Bone Miner Res. 1992; 7:1005–10. 10.1002/jbmr.5650070902 1414493

[2] Melton LJ 3rd, Atkinson EJ, O'Connor MK, O'Fallon WM, Riggs BL. Bone density and fracture risk in men. J Bone Miner Res. 1998; 13:1915–23. 10.1359/jbmr.1998.13.12.1915 9844110

[3] Kanis JA, Johnell O, Oden A, Sembo I, Redlund-Johnell I, Dawson A, De Laet C, Jonsson B. Long-term risk of osteoporotic fracture in Malmö. Osteoporos Int. 2000; 11:669–74. 10.1007/s001980070064 11095169

[4] Blume SW, Curtis JR. Medical costs of osteoporosis in the elderly Medicare population. Osteoporos Int. 2011; 22:1835–44. 10.1007/s00198-010-1419-7 21165602 PMC3767374

[5] Cauley JA, Wampler NS, Barnhart JM, Wu L, Allison M, Chen Z, Hendrix S, Robbins J, Jackson RD, and Women's Health Initiative Observational Study. Incidence of fractures compared to cardiovascular disease and breast cancer: the Women's Health Initiative Observational Study. Osteoporos Int. 2008; 19:1717–23. 10.1007/s00198-008-0634-y 18629572 PMC2663802

[6] Cauley JA. Public health impact of osteoporosis. J Gerontol A Biol Sci Med Sci. 2013; 68:1243–51. 10.1093/gerona/glt093 23902935 PMC3779634

[7] Khosla S, Hofbauer LC. Osteoporosis treatment: recent developments and ongoing challenges. Lancet Diabetes Endocrinol. 2017; 5:898–907. 10.1016/S2213-8587(17)30188-2 28689769 PMC5798872

[8] Ayturk UM, Jacobsen CM, Christodoulou DC, Gorham J, Seidman JG, Seidman CE, Robling AG, Warman ML. An RNA-seq protocol to identify mRNA expression changes in mouse diaphyseal bone: applications in mice with bone property altering Lrp5 mutations. J Bone Miner Res. 2013; 28:2081–93. 10.1002/jbmr.1946 23553928 PMC3743099

[9] Farr JN, Roforth MM, Fujita K, Nicks KM, Cunningham JM, Atkinson EJ, Therneau TM, McCready LK, Peterson JM, Drake MT, Monroe DG, Khosla S. Effects of Age and Estrogen on Skeletal Gene Expression in Humans as Assessed by RNA Sequencing. PLoS One. 2015; 10:e0138347. 10.1371/journal.pone.0138347 26402159 PMC4581624

[10] Kelly NH, Schimenti JC, Ross FP, van der Meulen MC. Transcriptional profiling of cortical versus cancellous bone from mechanically-loaded murine tibiae reveals differential gene expression. Bone. 2016; 86:22–9. 10.1016/j.bone.2016.02.007 26876048 PMC4833881

[11] Chermside-Scabbo CJ, Harris TL, Brodt MD, Braenne I, Zhang B, Farber CR, Silva MJ. Old Mice Have Less Transcriptional Activation But Similar Periosteal Cell Proliferation Compared to Young-Adult Mice in Response to in vivo Mechanical Loading. J Bone Miner Res. 2020; 35:1751–64. 10.1002/jbmr.4031 32311160 PMC7486279

[12] Hoffman JM, Lyu Y, Pletcher SD, Promislow DEL. Proteomics and metabolomics in ageing research: from biomarkers to systems biology. Essays Biochem. 2017; 61:379–88. 10.1042/EBC20160083 28698311 PMC5743054

[13] Foster LJ, Zeemann PA, Li C, Mann M, Jensen ON, Kassem M. Differential expression profiling of membrane proteins by quantitative proteomics in a human mesenchymal stem cell line undergoing osteoblast differentiation. Stem Cells. 2005; 23:1367–77. 10.1634/stemcells.2004-0372 16210410

[14] Choi YA, Lim J, Kim KM, Acharya B, Cho JY, Bae YC, Shin HI, Kim SY, Park EK. Secretome analysis of human BMSCs and identification of SMOC1 as an important ECM protein in osteoblast differentiation. J Proteome Res. 2010; 9:2946–56. 10.1021/pr901110q 20359165

[15] Govey PM, Jacobs JM, Tilton SC, Loiselle AE, Zhang Y, Freeman WM, Waters KM, Karin NJ, Donahue HJ. Integrative transcriptomic and proteomic analysis of osteocytic cells exposed to fluid flow reveals novel mechano-sensitive signaling pathways. J Biomech. 2014; 47:1838–45. 10.1016/j.jbiomech.2014.03.022 24720889 PMC4037855

[16] Zhang C, Xu S, Zhang S, Liu M, Du H, Sun R, Jing B, Sun Y. Ageing characteristics of bone indicated by transcriptomic and exosomal proteomic analysis of cortical bone cells. J Orthop Surg Res. 2019; 14:129. 10.1186/s13018-019-1163-4 31077243 PMC6509863

[17] Jiang X, Ye M, Jiang X, Liu G, Feng S, Cui L, Zou H. Method development of efficient protein extraction in bone tissue for proteome analysis. J Proteome Res. 2007; 6:2287–94. 10.1021/pr070056t 17488005

[18] Li J, Zhang F, Chen JY. An integrated proteomics analysis of bone tissues in response to mechanical stimulation. BMC Syst Biol. 2011 (Suppl 3); 5:S7. 10.1186/1752-0509-5-S3-S7 22784626 PMC3287575

[19] Zhang WB, Wang L. Label-free quantitative proteome analysis of skeletal tissues under mechanical load. J Cell Biochem. 2009; 108:600–11. 10.1002/jcb.22291 19670388

[20] Chaput CD, Dangott LJ, Rahm MD, Hitt KD, Stewart DS, Wayne Sampson H. A proteomic study of protein variation between osteopenic and age-matched control bone tissue. Exp Biol Med (Maywood). 2012; 237:491–8. 10.1258/ebm.2012.011374 22619369

[21] Wolff J. Das gesetz der transformation der knochen. A Hirshwald. 1892; 1:1–152.

[22] Frost HM. The laws of bone structure. Springfield, Ill.: Thomas. 1964.

[23] Korpelainen R, Keinänen-Kiukaanniemi S, Heikkinen J, Väänänen K, Korpelainen J. Effect of impact exercise on bone mineral density in elderly women with low BMD: a population-based randomized controlled 30-month intervention. Osteoporos Int. 2006; 17:109–18. 10.1007/s00198-005-1924-2 15889312

[24] Howe TE, Shea B, Dawson LJ, Downie F, Murray A, Ross C, Harbour RT, Caldwell LM, Creed G. Exercise for preventing and treating osteoporosis in postmenopausal women. Cochrane Database Syst Rev. 2011; CD000333. 10.1002/14651858.CD000333.pub2 21735380 PMC12744941

[25] Marques EA, Mota J, Carvalho J. Exercise effects on bone mineral density in older adults: a meta-analysis of randomized controlled trials. Age (Dordr). 2012; 34:1493–515. 10.1007/s11357-011-9311-8 21922251 PMC3528362

[26] Lynch ME, Main RP, Xu Q, Schmicker TL, Schaffler MB, Wright TM, van der Meulen MC. Tibial compression is anabolic in the adult mouse skeleton despite reduced responsiveness with aging. Bone. 2011; 49:439–46. 10.1016/j.bone.2011.05.017 21642027 PMC3235401

[27] Willie BM, Birkhold AI, Razi H, Thiele T, Aido M, Kruck B, Schill A, Checa S, Main RP, Duda GN. Diminished response to in vivo mechanical loading in trabecular and not cortical bone in adulthood of female C57Bl/6 mice coincides with a reduction in deformation to load. Bone. 2013; 55:335–46. 10.1016/j.bone.2013.04.023 23643681

[28] Holguin N, Brodt MD, Sanchez ME, Silva MJ. Aging diminishes lamellar and woven bone formation induced by tibial compression in adult C57BL/6. Bone. 2014; 65:83–91. 10.1016/j.bone.2014.05.006 24836737 PMC4091978

[29] Meakin LB, Galea GL, Sugiyama T, Lanyon LE, Price JS. Age-related impairment of bones' adaptive response to loading in mice is associated with sex-related deficiencies in osteoblasts but no change in osteocytes. J Bone Miner Res. 2014; 29:1859–71. 10.1002/jbmr.2222 24644060 PMC4258100

[30] Birkhold AI, Razi H, Duda GN, Weinkamer R, Checa S, Willie BM. The influence of age on adaptive bone formation and bone resorption. Biomaterials. 2014; 35:9290–301. 10.1016/j.biomaterials.2014.07.051 25128376

[31] Kosti I, Jain N, Aran D, Butte AJ, Sirota M. Cross-tissue Analysis of Gene and Protein Expression in Normal and Cancer Tissues. Sci Rep. 2016; 6:24799. 10.1038/srep24799 27142790 PMC4855174

[32] Wilhelm M, Schlegl J, Hahne H, Gholami AM, Lieberenz M, Savitski MM, Ziegler E, Butzmann L, Gessulat S, Marx H, Mathieson T, Lemeer S, Schnatbaum K, et al. Mass-spectrometry-based draft of the human proteome. Nature. 2014; 509:582–7. 10.1038/nature13319 24870543

[33] Kim SK. Identification of 613 new loci associated with heel bone mineral density and a polygenic risk score for bone mineral density, osteoporosis and fracture. PLoS One. 2018; 13:e0200785. 10.1371/journal.pone.0200785 30048462 PMC6062019

[34] Liu J, Zhou Y, Liu S, Song X, Yang XZ, Fan Y, Chen W, Akdemir ZC, Yan Z, Zuo Y, Du R, Liu Z, Yuan B, et al, and DISCO (Deciphering disorders Involving Scoliosis and COmorbidities) Study. The coexistence of copy number variations (CNVs) and single nucleotide polymorphisms (SNPs) at a locus can result in distorted calculations of the significance in associating SNPs to disease. Hum Genet. 2018; 137:553–67. 10.1007/s00439-018-1910-3 30019117 PMC6200315

[35] Chesi A, Mitchell JA, Kalkwarf HJ, Bradfield JP, Lappe JM, Cousminer DL, Roy SM, McCormack SE, Gilsanz V, Oberfield SE, Hakonarson H, Shepherd JA, Kelly A, et al. A Genomewide Association Study Identifies Two Sex-Specific Loci, at SPTB and IZUMO3, Influencing Pediatric Bone Mineral Density at Multiple Skeletal Sites. J Bone Miner Res. 2017; 32:1274–81. 10.1002/jbmr.3097 28181694 PMC5466475

[36] Kichaev G, Bhatia G, Loh PR, Gazal S, Burch K, Freund MK, Schoech A, Pasaniuc B, Price AL. Leveraging Polygenic Functional Enrichment to Improve GWAS Power. Am J Hum Genet. 2019; 104:65–75. 10.1016/j.ajhg.2018.11.008 30595370 PMC6323418

[37] Styrkarsdottir U, Halldorsson BV, Gretarsdottir S, Gudbjartsson DF, Walters GB, Ingvarsson T, Jonsdottir T, Saemundsdottir J, Snorradóttir S, Center JR, Nguyen TV, Alexandersen P, Gulcher JR, et al. New sequence variants associated with bone mineral density. Nat Genet. 2009; 41:15–7. 10.1038/ng.284 19079262

[38] Estrada K, Styrkarsdottir U, Evangelou E, Hsu YH, Duncan EL, Ntzani EE, Oei L, Albagha OM, Amin N, Kemp JP, Koller DL, Li G, Liu CT, et al. Genome-wide meta-analysis identifies 56 bone mineral density loci and reveals 14 loci associated with risk of fracture. Nat Genet. 2012; 44:491–501. 10.1038/ng.2249 22504420 PMC3338864

[39] Morris JA, Kemp JP, Youlten SE, Laurent L, Logan JG, Chai RC, Vulpescu NA, Forgetta V, Kleinman A, Mohanty ST, Sergio CM, Quinn J, Nguyen-Yamamoto L, et al, and 23andMe Research Team. An atlas of genetic influences on osteoporosis in humans and mice. Nat Genet. 2019; 51:258–66. 10.1038/s41588-018-0302-x 30598549 PMC6358485

[40] Gudbjartsson DF, Walters GB, Thorleifsson G, Stefansson H, Halldorsson BV, Zusmanovich P, Sulem P, Thorlacius S, Gylfason A, Steinberg S, Helgadottir A, Ingason A, Steinthorsdottir V, et al. Many sequence variants affecting diversity of adult human height. Nat Genet. 2008; 40:609–15. 10.1038/ng.122 18391951

[41] Richardson TG, Sanderson E, Elsworth B, Tilling K, Davey Smith G. Use of genetic variation to separate the effects of early and later life adiposity on disease risk: mendelian randomisation study. BMJ. 2020; 369:m1203. 10.1136/bmj.m1203 32376654 PMC7201936

[42] Tachmazidou I, Süveges D, Min JL, Ritchie GRS, Steinberg J, Walter K, Iotchkova V, Schwartzentruber J, Huang J, Memari Y, McCarthy S, Crawford AA, Bombieri C, et al, and SpiroMeta Consortium, and GoT2D Consortium, and arcOGEN Consortium, and Understanding Society Scientific Group, and UK10K Consortium. Whole-Genome Sequencing Coupled to Imputation Discovers Genetic Signals for Anthropometric Traits. Am J Hum Genet. 2017; 100:865–84. 10.1016/j.ajhg.2017.04.014 28552196 PMC5473732

[43] Styrkarsdottir U, Stefansson OA, Gunnarsdottir K, Thorleifsson G, Lund SH, Stefansdottir L, Juliusson K, Agustsdottir AB, Zink F, Halldorsson GH, Ivarsdottir EV, Benonisdottir S, Jonsson H, et al. GWAS of bone size yields twelve loci that also affect height, BMD, osteoarthritis or fractures. Nat Commun. 2019; 10:2054. 10.1038/s41467-019-09860-0 31053729 PMC6499783

[44] Berndt SI, Gustafsson S, Mägi R, Ganna A, Wheeler E, Feitosa MF, Justice AE, Monda KL, Croteau-Chonka DC, Day FR, Esko T, Fall T, Ferreira T, et al. Genome-wide meta-analysis identifies 11 new loci for anthropometric traits and provides insights into genetic architecture. Nat Genet. 2013; 45:501–12. 10.1038/ng.2606 23563607 PMC3973018

[45] Kemp JP, Morris JA, Medina-Gomez C, Forgetta V, Warrington NM, Youlten SE, Zheng J, Gregson CL, Grundberg E, Trajanoska K, Logan JG, Pollard AS, Sparkes PC, et al. Identification of 153 new loci associated with heel bone mineral density and functional involvement of GPC6 in osteoporosis. Nat Genet. 2017; 49:1468–75. 10.1038/ng.3949 28869591 PMC5621629

[46] Kim JJ, Lee HI, Park T, Kim K, Lee JE, Cho NH, Shin C, Cho YS, Lee JY, Han BG, Yoo HW, Lee JK. Identification of 15 loci influencing height in a Korean population. J Hum Genet. 2010; 55:27–31. 10.1038/jhg.2009.116 19893584

[47] Croteau-Chonka DC, Marvelle AF, Lange EM, Lee NR, Adair LS, Lange LA, Mohlke KL. Genome-wide association study of anthropometric traits and evidence of interactions with age and study year in Filipino women. Obesity (Silver Spring). 2011; 19:1019–27. 10.1038/oby.2010.256 20966902 PMC3046220

[48] Rüeger S, McDaid A, Kutalik Z. Evaluation and application of summary statistic imputation to discover new height-associated loci. PLoS Genet. 2018; 14:e1007371. 10.1371/journal.pgen.1007371 29782485 PMC5983877

[49] Akiyama M, Ishigaki K, Sakaue S, Momozawa Y, Horikoshi M, Hirata M, Matsuda K, Ikegawa S, Takahashi A, Kanai M, Suzuki S, Matsui D, Naito M, et al. Characterizing rare and low-frequency height-associated variants in the Japanese population. Nat Commun. 2019; 10:4393. 10.1038/s41467-019-12276-5 31562340 PMC6764965

[50] Wright KM, Rand KA, Kermany A, Noto K, Curtis D, Garrigan D, Slinkov D, Dorfman I, Granka JM, Byrnes J, Myres N, Ball CA, Ruby JG. A Prospective Analysis of Genetic Variants Associated with Human Lifespan. G3 (Bethesda). 2019; 9:2863–78. 10.1534/g3.119.400448 31484785 PMC6723124

[51] Roberts V, Main B, Timpson NJ, Haworth S. Genome-Wide Association Study Identifies Genetic Associations with Perceived Age. J Invest Dermatol. 2020; 140:2380–5. 10.1016/j.jid.2020.03.970 32339537 PMC7685007

[52] Tang SY, Herber RP, Ho SP, Alliston T. Matrix metalloproteinase-13 is required for osteocytic perilacunar remodeling and maintains bone fracture resistance. J Bone Miner Res. 2012; 27:1936–50. 10.1002/jbmr.1646 22549931 PMC3415585

[53] Mazur CM, Woo JJ, Yee CS, Fields AJ, Acevedo C, Bailey KN, Kaya S, Fowler TW, Lotz JC, Dang A, Kuo AC, Vail TP, Alliston T. Osteocyte dysfunction promotes osteoarthritis through MMP13-dependent suppression of subchondral bone homeostasis. Bone Res. 2019; 7:34. 10.1038/s41413-019-0070-y 31700695 PMC6828661

[54] Rauner M, Föger-Samwald U, Kurz MF, Brünner-Kubath C, Schamall D, Kapfenberger A, Varga P, Kudlacek S, Wutzl A, Höger H, Zysset PK, Shi GP, Hofbauer LC, et al. Cathepsin S controls adipocytic and osteoblastic differentiation, bone turnover, and bone microarchitecture. Bone. 2014; 64:281–7. 10.1016/j.bone.2014.04.022 24780878

[55] Reddy S, Devlin R, Menaa C, Nishimura R, Choi SJ, Dallas M, Yoneda T, Roodman GD. Isolation and characterization of a cDNA clone encoding a novel peptide (OSF) that enhances osteoclast formation and bone resorption. J Cell Physiol. 1998; 177:636–45. 10.1002/(SICI)1097-4652(199812)177:4<636::AID-JCP14>3.0.CO;2-H 10092216

[56] Kevorkova O, Martineau C, Martin-Falstrault L, Sanchez-Dardon J, Brissette L, Moreau R. Low-bone-mass phenotype of deficient mice for the cluster of differentiation 36 (CD36). PLoS One. 2013; 8:e77701. 10.1371/journal.pone.0077701 24204923 PMC3808405

[57] Koduru SV, Sun BH, Walker JM, Zhu M, Simpson C, Dhodapkar M, Insogna KL. The contribution of cross-talk between the cell-surface proteins CD36 and CD47-TSP-1 in osteoclast formation and function. J Biol Chem. 2018; 293:15055–69. 10.1074/jbc.RA117.000633 30082316 PMC6166722

[58] Middel V, Zhou L, Takamiya M, Beil T, Shahid M, Roostalu U, Grabher C, Rastegar S, Reischl M, Nienhaus GU, Strähle U. Dysferlin-mediated phosphatidylserine sorting engages macrophages in sarcolemma repair. Nat Commun. 2016; 7:12875. 10.1038/ncomms12875 27641898 PMC5031802

[59] Johnson CP. Emerging Functional Differences between the Synaptotagmin and Ferlin Calcium Sensor Families. Biochemistry. 2017; 56:6413–7. 10.1021/acs.biochem.7b00928 29110470 PMC5730944

[60] Saag KG, Petersen J, Brandi ML, Karaplis AC, Lorentzon M, Thomas T, Maddox J, Fan M, Meisner PD, Grauer A. Romosozumab or Alendronate for Fracture Prevention in Women with Osteoporosis. N Engl J Med. 2017; 377:1417–27. 10.1056/NEJMoa1708322 28892457

[61] Bilen MA, Pan T, Lee YC, Lin SC, Yu G, Pan J, Hawke D, Pan BF, Vykoukal J, Gray K, Satcher RL, Gallick GE, Yu-Lee LY, Lin SH. Proteomics Profiling of Exosomes from Primary Mouse Osteoblasts under Proliferation versus Mineralization Conditions and Characterization of Their Uptake into Prostate Cancer Cells. J Proteome Res. 2017; 16:2709–28. 10.1021/acs.jproteome.6b00981 28675788 PMC5860883

[62] Kahai S, Vary CP, Gao Y, Seth A. Collagen, type V, alpha1 (COL5A1) is regulated by TGF-beta in osteoblasts. Matrix Biol. 2004; 23:445–55. 10.1016/j.matbio.2004.09.004 15579311

[63] Hafez A, Squires R, Pedracini A, Joshi A, Seegmiller RE, Oxford JT. Col11a1 Regulates Bone Microarchitecture during Embryonic Development. J Dev Biol. 2015; 3:158–76. 10.3390/jdb3040158 26779434 PMC4711924

[64] Hayer S, Steiner G, Görtz B, Reiter E, Tohidast-Akrad M, Amling M, Hoffmann O, Redlich K, Zwerina J, Skriner K, Hilberg F, Wagner EF, Smolen JS, Schett G. CD44 is a determinant of inflammatory bone loss. J Exp Med. 2005; 201:903–14. 10.1084/jem.20040852 15781582 PMC2213110

[65] Dong L, Wu J, Chen K, Xie J, Wang Y, Li D, Liu Y, Yin A, Zhao Y, Han Y, Zhou J, Zhang L, Chen Z, Zuo D. Mannan-Binding Lectin Attenuates Inflammatory Arthritis Through the Suppression of Osteoclastogenesis. Front Immunol. 2019; 10:1239. 10.3389/fimmu.2019.01239 31214191 PMC6557994

[66] Tucker RP, Degen M. The Expression and Possible Functions of Tenascin-W During Development and Disease. Front Cell Dev Biol. 2019; 7:53. 10.3389/fcell.2019.00053 31032255 PMC6473177

[67] Morgan JM, Wong A, Yellowley CE, Genetos DC. Regulation of tenascin expression in bone. J Cell Biochem. 2011; 112:3354–63. 10.1002/jcb.23265 21751239 PMC3196820

[68] Ker DFE, Wang D, Sharma R, Zhang B, Passarelli B, Neff N, Li C, Maloney W, Quake S, Yang YP. Identifying deer antler uhrf1 proliferation and s100a10 mineralization genes using comparative RNA-seq. Stem Cell Res Ther. 2018; 9:292. 10.1186/s13287-018-1027-6 30376879 PMC6208050

[69] Guérit D, Marie P, Morel A, Maurin J, Verollet C, Raynaud-Messina B, Urbach S, Blangy A. Primary myeloid cell proteomics and transcriptomics: importance of β-tubulin isotypes for osteoclast function. J Cell Sci. 2020; 133:jcs239772. 10.1242/jcs.239772 32265273

[70] Sztacho M, Segeletz S, Sanchez-Fernandez MA, Czupalla C, Niehage C, Hoflack B. BAR Proteins PSTPIP1/2 Regulate Podosome Dynamics and the Resorption Activity of Osteoclasts. PLoS One. 2016; 11:e0164829. 10.1371/journal.pone.0164829 27760174 PMC5070766

[71] Vives V, Laurin M, Cres G, Larrousse P, Morichaud Z, Noel D, Côté JF, Blangy A. The Rac1 exchange factor Dock5 is essential for bone resorption by osteoclasts. J Bone Miner Res. 2011; 26:1099–110. 10.1002/jbmr.282 21542010 PMC4640905

[72] Vives V, Cres G, Richard C, Busson M, Ferrandez Y, Planson AG, Zeghouf M, Cherfils J, Malaval L, Blangy A. Pharmacological inhibition of Dock5 prevents osteolysis by affecting osteoclast podosome organization while preserving bone formation. Nat Commun. 2015; 6:6218. 10.1038/ncomms7218 25645278

[73] Paloneva J, Kestilä M, Wu J, Salminen A, Böhling T, Ruotsalainen V, Hakola P, Bakker AB, Phillips JH, Pekkarinen P, Lanier LL, Timonen T, Peltonen L. Loss-of-function mutations in TYROBP (DAP12) result in a presenile dementia with bone cysts. Nat Genet. 2000; 25:357–61. 10.1038/77153 10888890

[74] Heymann D, Guicheux J, Gouin F, Passuti N, Daculsi G. Cytokines, growth factors and osteoclasts. Cytokine. 1998; 10:155–68. 10.1006/cyto.1997.0277 9576060

[75] Tominaga H, Maeda S, Hayashi M, Takeda S, Akira S, Komiya S, Nakamura T, Akiyama H, Imamura T. CCAAT/enhancer-binding protein beta promotes osteoblast differentiation by enhancing Runx2 activity with ATF4. Mol Biol Cell. 2008; 19:5373–86. 10.1091/mbc.e08-03-0329 18843047 PMC2592674

[76] Sabir HJ, Nehlin JO, Qanie D, Harkness L, Prokhorova TA, Blagoev B, Kassem M, Isa A, Barington T. Separate developmental programs for HLA-A and -B cell surface expression during differentiation from embryonic stem cells to lymphocytes, adipocytes and osteoblasts. PLoS One. 2013; 8:e54366. 10.1371/journal.pone.0054366 23349864 PMC3548781

[77] Kim JH, Kim K, Youn BU, Jin HM, Kim N. MHC class II transactivator negatively regulates RANKL-mediated osteoclast differentiation by downregulating NFATc1 and OSCAR. Cell Signal. 2010; 22:1341–9. 10.1016/j.cellsig.2010.05.001 20466061

[78] Nakano Y, Toyosawa S, Takano Y. Eccentric localization of osteocytes expressing enzymatic activities, protein, and mRNA signals for type 5 tartrate-resistant acid phosphatase (TRAP). J Histochem Cytochem. 2004; 52:1475–82. 10.1369/jhc.4A6378.2004 15505342 PMC3957824

[79] Jin Z, Wei W, Huynh H, Wan Y. HDAC9 Inhibits Osteoclastogenesis via Mutual Suppression of PPARγ/RANKL Signaling. Mol Endocrinol. 2015; 29:730–8. 10.1210/me.2014-1365 25793404 PMC4415206

[80] Dallas SL, Rosser JL, Mundy GR, Bonewald LF. Proteolysis of latent transforming growth factor-beta (TGF-beta )-binding protein-1 by osteoclasts. A cellular mechanism for release of TGF-beta from bone matrix. J Biol Chem. 2002; 277:21352–60. 10.1074/jbc.M111663200 11929865

[81] Tsukamoto S, Mizuta T, Fujimoto M, Ohte S, Osawa K, Miyamoto A, Yoneyama K, Murata E, Machiya A, Jimi E, Kokabu S, Katagiri T. Smad9 is a new type of transcriptional regulator in bone morphogenetic protein signaling. Sci Rep. 2014; 4:7596. 10.1038/srep07596 25534700 PMC4274517

[82] Liu T, Li B, Zheng XF, Jiang SD, Zhou ZZ, Xu WN, Zheng HL, Wang CD, Zhang XL, Jiang LS. Chordin-Like 1 Improves Osteogenesis of Bone Marrow Mesenchymal Stem Cells Through Enhancing BMP4-SMAD Pathway. Front Endocrinol (Lausanne). 2019; 10:360. 10.3389/fendo.2019.00360 31249554 PMC6582276

[83] Oichi T, Taniguchi Y, Soma K, Oshima Y, Yano F, Mori Y, Chijimatsu R, Kim-Kaneyama JR, Tanaka S, Saito T. Adamts17 is involved in skeletogenesis through modulation of BMP-Smad1/5/8 pathway. Cell Mol Life Sci. 2019; 76:4795–809. 10.1007/s00018-019-03188-0 31201465 PMC11105417

[84] Abstracts from the Bone and Muscle Interactions: The Mechanical and Beyond Meeting August 2019. JBMR Plus. 2019; 3:e10257. 10.1002/jbm4.10257

[85] Moorer MC, Riddle RC. Regulation of Osteoblast Metabolism by Wnt Signaling. Endocrinol Metab (Seoul). 2018; 33:318–30. 10.3803/EnM.2018.33.3.318 30112869 PMC6145954

[86] Lee YS, Chuong CM. Adhesion molecules in skeletogenesis: I. Transient expression of neural cell adhesion molecules (NCAM) in osteoblasts during endochondral and intramembranous ossification. J Bone Miner Res. 1992; 7:1435–46. 10.1002/jbmr.5650071211 1481729

[87] Yan YX, Gong YW, Guo Y, Lv Q, Guo C, Zhuang Y, Zhang Y, Li R, Zhang XZ. Mechanical strain regulates osteoblast proliferation through integrin-mediated ERK activation. PLoS One. 2012; 7:e35709. 10.1371/journal.pone.0035709 22539993 PMC3335094

[88] Coudert AE, Del Fattore A, Baulard C, Olaso R, Schiltz C, Collet C, Teti A, de Vernejoul MC. Differentially expressed genes in autosomal dominant osteopetrosis type II osteoclasts reveal known and novel pathways for osteoclast biology. Lab Invest. 2014; 94:275–85. 10.1038/labinvest.2013.140 24336069

[89] Dole NS, Mazur CM, Acevedo C, Lopez JP, Monteiro DA, Fowler TW, Gludovatz B, Walsh F, Regan JN, Messina S, Evans DS, Lang TF, Zhang B, et al. Osteocyte-Intrinsic TGF-β Signaling Regulates Bone Quality through Perilacunar/Canalicular Remodeling. Cell Rep. 2017; 21:2585–96. 10.1016/j.celrep.2017.10.115 29186693 PMC6014615

[90] Marchesini N, Hannun YA. Acid and neutral sphingomyelinases: roles and mechanisms of regulation. Biochem Cell Biol. 2004; 82:27–44. 10.1139/o03-091 15052326

[91] Ruvolo PP. Ceramide regulates cellular homeostasis via diverse stress signaling pathways. Leukemia. 2001; 15:1153–60. 10.1038/sj.leu.2402197 11480555

[92] Li J, Manickam G, Ray S, Oh CD, Yasuda H, Moffatt P, Murshed M. Smpd3 Expression in both Chondrocytes and Osteoblasts Is Required for Normal Endochondral Bone Development. Mol Cell Biol. 2016; 36:2282–99. 10.1128/MCB.01077-15 27325675 PMC4985927

[93] Manickam G, Moffatt P, Murshed M. Role of SMPD3 during Bone Fracture Healing and Regulation of Its Expression. Mol Cell Biol. 2019; 39:e00370–18. 10.1128/MCB.00370-18 30530524 PMC6362318

[94] Harris TL, Silva MJ. Gene expression of intracortical bone demonstrates loading-induced increases in Wnt1 and Ngf and inhibition of bone remodeling processes. Bone. 2021; 150:116019. 10.1016/j.bone.2021.116019 34023542 PMC8408835

[95] GTEx Consortium. The Genotype-Tissue Expression (GTEx) project. Nat Genet. 2013; 45:580–5. 10.1038/ng.2653 23715323 PMC4010069

[96] Kim MS, Pinto SM, Getnet D, Nirujogi RS, Manda SS, Chaerkady R, Madugundu AK, Kelkar DS, Isserlin R, Jain S, Thomas JK, Muthusamy B, Leal-Rojas P, et al. A draft map of the human proteome. Nature. 2014; 509:575–81. 10.1038/nature13302 24870542 PMC4403737

[97] Gould NR, Williams KM, Joca HC, Torre OM, Lyons JS, Leser JM, Srikanth MP, Hughes M, Khairallah RJ, Feldman RA, Ward CW, Stains JP. Disparate bone anabolic cues activate bone formation by regulating the rapid lysosomal degradation of sclerostin protein. Elife. 2021; 10:e64393. 10.7554/eLife.64393 33779549 PMC8032393

[98] de Sousa Abreu R, Penalva LO, Marcotte EM, Vogel C. Global signatures of protein and mRNA expression levels. Mol Biosyst. 2009; 5:1512–26. 10.1039/b908315d 20023718 PMC4089977

[99] Liu Y, Beyer A, Aebersold R. On the Dependency of Cellular Protein Levels on mRNA Abundance. Cell. 2016; 165:535–50. 10.1016/j.cell.2016.03.014 27104977

[100] López-Otín C, Blasco MA, Partridge L, Serrano M, Kroemer G. The hallmarks of aging. Cell. 2013; 153:1194–217. 10.1016/j.cell.2013.05.039 23746838 PMC3836174

[101] Kaushik S, Cuervo AM. Proteostasis and aging. Nat Med. 2015; 21:1406–15. 10.1038/nm.4001 26646497

[102] Farr JN, Almeida M. The Spectrum of Fundamental Basic Science Discoveries Contributing to Organismal Aging. J Bone Miner Res. 2018; 33:1568–84. 10.1002/jbmr.3564 30075061 PMC6327947

[103] Guo D, Keightley A, Guthrie J, Veno PA, Harris SE, Bonewald LF. Identification of osteocyte-selective proteins. Proteomics. 2010; 10:3688–98. 10.1002/pmic.201000306 20845334 PMC3517134

[104] Jilka RL, O'Brien CA. The Role of Osteocytes in Age-Related Bone Loss. Curr Osteoporos Rep. 2016; 14:16–25. 10.1007/s11914-016-0297-0 26909563

[105] Tiede-Lewis LM, Xie Y, Hulbert MA, Campos R, Dallas MR, Dusevich V, Bonewald LF, Dallas SL. Degeneration of the osteocyte network in the C57BL/6 mouse model of aging. Aging (Albany NY). 2017; 9:2190–208. 10.18632/aging.101308 29074822 PMC5680562

[106] Noble BS, Peet N, Stevens HY, Brabbs A, Mosley JR, Reilly GC, Reeve J, Skerry TM, Lanyon LE. Mechanical loading: biphasic osteocyte survival and targeting of osteoclasts for bone destruction in rat cortical bone. Am J Physiol Cell Physiol. 2003; 284:C934–43. 10.1152/ajpcell.00234.2002 12477665

[107] Ross JM, Coppotelli G, Branca RM, Kim KM, Lehtiö J, Sinclair DA, Olson L. Voluntary exercise normalizes the proteomic landscape in muscle and brain and improves the phenotype of progeroid mice. Aging Cell. 2019; 18:e13029. 10.1111/acel.13029 31489782 PMC6826127

[108] Vidal C, Bermeo S, Fatkin D, Duque G. Role of the nuclear envelope in the pathogenesis of age-related bone loss and osteoporosis. Bonekey Rep. 2012; 1:62. 10.1038/bonekey.2012.62 23951459 PMC3727739

[109] Dahl KN, Ribeiro AJ, Lammerding J. Nuclear shape, mechanics, and mechanotransduction. Circ Res. 2008; 102:1307–18. 10.1161/CIRCRESAHA.108.173989 18535268 PMC2717705

[110] Dew G, Murphy G, Stanton H, Vallon R, Angel P, Reynolds JJ, Hembry RM. Localisation of matrix metalloproteinases and TIMP-2 in resorbing mouse bone. Cell Tissue Res. 2000; 299:385–94. 10.1007/s004419900166 10772252

[111] Hayakawa T. Multiple functions of tissue inhibitors of metalloproteinases (TIMPs): a new aspect involving osteoclastic bone resorption. J Bone Miner Metab. 2002; 20:1–13. 10.1007/s774-002-8440-0 11810410

[112] Pelton RW, Saxena B, Jones M, Moses HL, Gold LI. Immunohistochemical localization of TGF beta 1, TGF beta 2, and TGF beta 3 in the mouse embryo: expression patterns suggest multiple roles during embryonic development. J Cell Biol. 1991; 115:1091–105. 10.1083/jcb.115.4.1091 1955457 PMC2289937

[113] Sanford LP, Ormsby I, Gittenberger-de Groot AC, Sariola H, Friedman R, Boivin GP, Cardell EL, Doetschman T. TGFbeta2 knockout mice have multiple developmental defects that are non-overlapping with other TGFbeta knockout phenotypes. Development. 1997; 124:2659–70. 10.1242/dev.124.13.2659 9217007 PMC3850286

[114] Li J, Ayoub A, Xiu Y, Yin X, Sanders JO, Mesfin A, Xing L, Yao Z, Boyce BF. TGFβ-induced degradation of TRAF3 in mesenchymal progenitor cells causes age-related osteoporosis. Nat Commun. 2019; 10:2795. 10.1038/s41467-019-10677-0 31243287 PMC6595054

[115] Robling AG, Niziolek PJ, Baldridge LA, Condon KW, Allen MR, Alam I, Mantila SM, Gluhak-Heinrich J, Bellido TM, Harris SE, Turner CH. Mechanical stimulation of bone in vivo reduces osteocyte expression of Sost/sclerostin. J Biol Chem. 2008; 283:5866–75. 10.1074/jbc.M705092200 18089564

[116] Thompson WR, Rubin CT, Rubin J. Mechanical regulation of signaling pathways in bone. Gene. 2012; 503:179–93. 10.1016/j.gene.2012.04.076 22575727 PMC3371109

[117] Overmyer KA, Evans CR, Qi NR, Minogue CE, Carson JJ, Chermside-Scabbo CJ, Koch LG, Britton SL, Pagliarini DJ, Coon JJ, Burant CF. Maximal oxidative capacity during exercise is associated with skeletal muscle fuel selection and dynamic changes in mitochondrial protein acetylation. Cell Metab. 2015; 21:468–78. 10.1016/j.cmet.2015.02.007 25738461 PMC4350023

[118] Dieterich DC, Link AJ, Graumann J, Tirrell DA, Schuman EM. Selective identification of newly synthesized proteins in mammalian cells using bioorthogonal noncanonical amino acid tagging (BONCAT). Proc Natl Acad Sci U S A. 2006; 103:9482–7. 10.1073/pnas.0601637103 16769897 PMC1480433

[119] Savitski MM, Mathieson T, Zinn N, Sweetman G, Doce C, Becher I, Pachl F, Kuster B, Bantscheff M. Measuring and managing ratio compression for accurate iTRAQ/TMT quantification. J Proteome Res. 2013; 12:3586–98. 10.1021/pr400098r 23768245

[120] Pappireddi N, Martin L, Wühr M. A Review on Quantitative Multiplexed Proteomics. Chembiochem. 2019; 20:1210–24. 10.1002/cbic.201800650 30609196 PMC6520187

[121] Erickson BK, Rose CM, Braun CR, Erickson AR, Knott J, McAlister GC, Wühr M, Paulo JA, Everley RA, Gygi SP. A Strategy to Combine Sample Multiplexing with Targeted Proteomics Assays for High-Throughput Protein Signature Characterization. Mol Cell. 2017; 65:361–70. 10.1016/j.molcel.2016.12.005 28065596 PMC5250569

[122] Meakin LB, Sugiyama T, Galea GL, Browne WJ, Lanyon LE, Price JS. Male mice housed in groups engage in frequent fighting and show a lower response to additional bone loading than females or individually housed males that do not fight. Bone. 2013; 54:113–7. 10.1016/j.bone.2013.01.029 23356987 PMC3607215

[123] Sun D, Brodt MD, Zannit HM, Holguin N, Silva MJ. Evaluation of loading parameters for murine axial tibial loading: Stimulating cortical bone formation while reducing loading duration. J Orthop Res. 2018; 36:682–91. 10.1002/jor.23727 28888055 PMC5839947

[124] Patel TK, Brodt MD, Silva MJ. Experimental and finite element analysis of strains induced by axial tibial compression in young-adult and old female C57Bl/6 mice. J Biomech. 2014; 47:451–7. 10.1016/j.jbiomech.2013.10.052 24268312 PMC3902696

[125] Holguin N, Brodt MD, Silva MJ. Activation of Wnt Signaling by Mechanical Loading Is Impaired in the Bone of Old Mice. J Bone Miner Res. 2016; 31:2215–26. 10.1002/jbmr.2900 27357062 PMC5397287

[126] Kelly NH, Schimenti JC, Patrick Ross F, van der Meulen MC. A method for isolating high quality RNA from mouse cortical and cancellous bone. Bone. 2014; 68:1–5. 10.1016/j.bone.2014.07.022 25073031 PMC4281890

[127] Mertins P, Yang F, Liu T, Mani DR, Petyuk VA, Gillette MA, Clauser KR, Qiao JW, Gritsenko MA, Moore RJ, Levine DA, Townsend R, Erdmann-Gilmore P, et al. Ischemia in tumors induces early and sustained phosphorylation changes in stress kinase pathways but does not affect global protein levels. Mol Cell Proteomics. 2014; 13:1690–704. 10.1074/mcp.M113.036392 24719451 PMC4083109

[128] Wiśniewski JR, Zougman A, Nagaraj N, Mann M. Universal sample preparation method for proteome analysis. Nat Methods. 2009; 6:359–62. 10.1038/nmeth.1322 19377485

[129] Chen ZW, Fuchs K, Sieghart W, Townsend RR, Evers AS. Deep amino acid sequencing of native brain GABAA receptors using high-resolution mass spectrometry. Mol Cell Proteomics. 2012; 11:M111.011445. 10.1074/mcp.M111.011445 22338125 PMC3270104

[130] Ritchie ME, Phipson B, Wu D, Hu Y, Law CW, Shi W, Smyth GK. limma powers differential expression analyses for RNA-sequencing and microarray studies. Nucleic Acids Res. 2015; 43:e47. 10.1093/nar/gkv007 25605792 PMC4402510

[131] Dobin A, Davis CA, Schlesinger F, Drenkow J, Zaleski C, Jha S, Batut P, Chaisson M, Gingeras TR. STAR: ultrafast universal RNA-seq aligner. Bioinformatics. 2013; 29:15–21. 10.1093/bioinformatics/bts635 23104886 PMC3530905

[132] Taverna F, Goveia J, Karakach TK, Khan S, Rohlenova K, Treps L, Subramanian A, Schoonjans L, Dewerchin M, Eelen G, Carmeliet P. BIOMEX: an interactive workflow for (single cell) omics data interpretation and visualization. Nucleic Acids Res. 2020; 48:W385–94. 10.1093/nar/gkaa332 32392297 PMC7319461

[133] Robinson MD, McCarthy DJ, Smyth GK. edgeR: a Bioconductor package for differential expression analysis of digital gene expression data. Bioinformatics. 2010; 26:139–40. 10.1093/bioinformatics/btp616 19910308 PMC2796818

[134] Liu R, Holik AZ, Su S, Jansz N, Chen K, Leong HS, Blewitt ME, Asselin-Labat ML, Smyth GK, Ritchie ME. Why weight? Modelling sample and observational level variability improves power in RNA-seq analyses. Nucleic Acids Res. 2015; 43:e97. 10.1093/nar/gkv412 25925576 PMC4551905

[135] Langfelder P, Horvath S. WGCNA: an R package for weighted correlation network analysis. BMC Bioinformatics. 2008; 9:559. 10.1186/1471-2105-9-559 19114008 PMC2631488

[136] Thomas PD, Campbell MJ, Kejariwal A, Mi H, Karlak B, Daverman R, Diemer K, Muruganujan A, Narechania A. PANTHER: a library of protein families and subfamilies indexed by function. Genome Res. 2003; 13:2129–41. 10.1101/gr.772403 12952881 PMC403709

[137] He L, Kernogitski Y, Kulminskaya I, Loika Y, Arbeev KG, Loiko E, Bagley O, Duan M, Yashkin A, Ukraintseva SV, Kovtun M, Yashin AI, Kulminski AM. Pleiotropic Meta-Analyses of Longitudinal Studies Discover Novel Genetic Variants Associated with Age-Related Diseases. Front Genet. 2016; 7:179. 10.3389/fgene.2016.00179 27790247 PMC5061751

