# Supplementary material for: A proteomics approach to study mouse long bones: examining baseline differences and mechanical loading-induced bone formation in young-adult and old mice
Source: Aging (Albany NY). 2024 Oct 12;16(19):12726–68. doi: 10.18632/aging.206131 (PMC11501390; doi:10.18632/aging.206131)
Supplement: Supplementary Figures [file aging-16-206131-s001.pdf]

SUPPLEMENTARY FIGURES

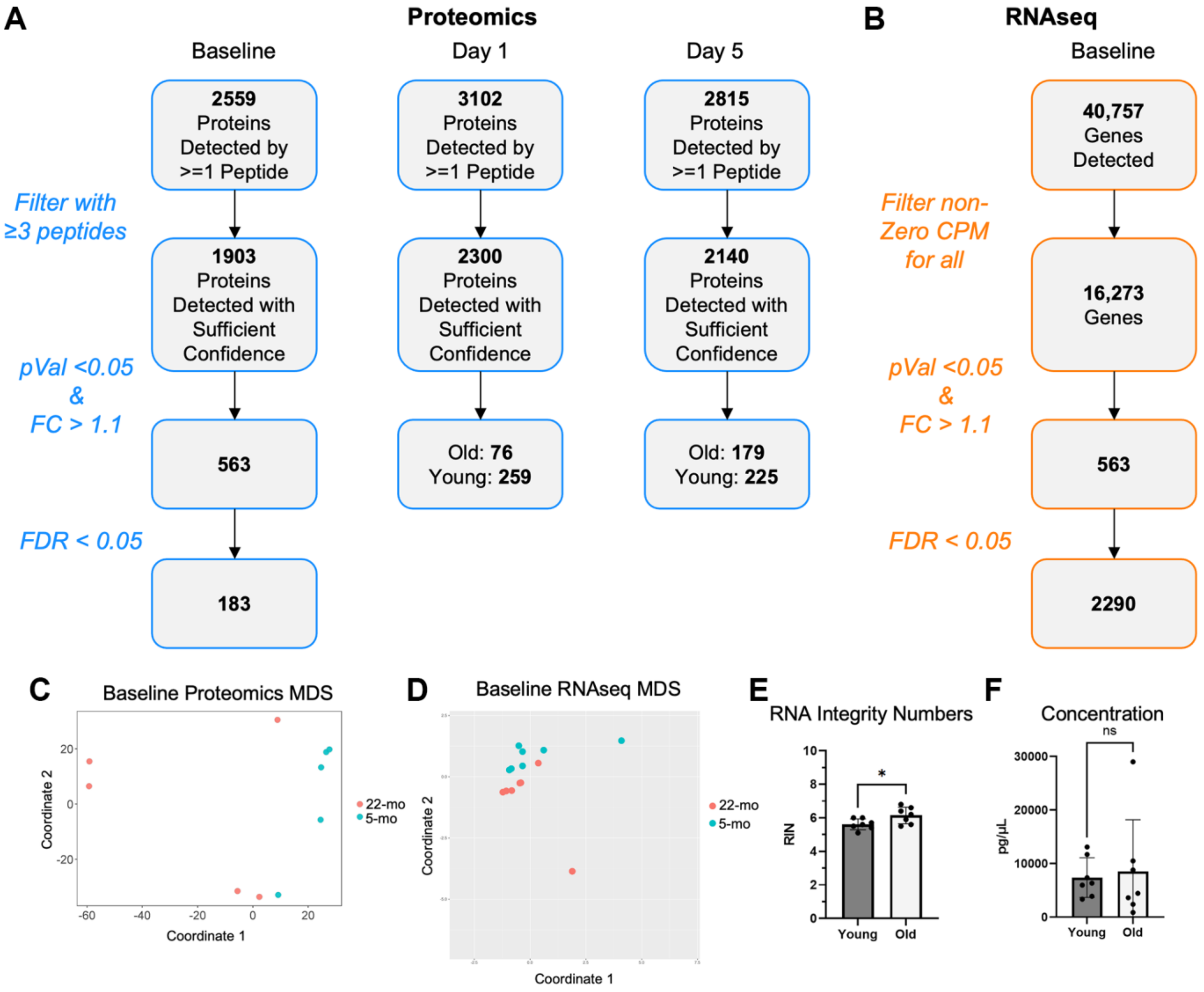

Supplementary Figure 1. Protein and gene filtering pipeline to define DEPs/DEGs, baseline MDS plots, and RNA information. (**A**) Proteomics filtering pipeline. (**B**) RNAseq filtering pipeline. (**C**) Baseline proteomics MDS. (**D**) Baseline RNAseq MDS. (**E**) RNA integrity numbers. (**F**) RNA concentration.

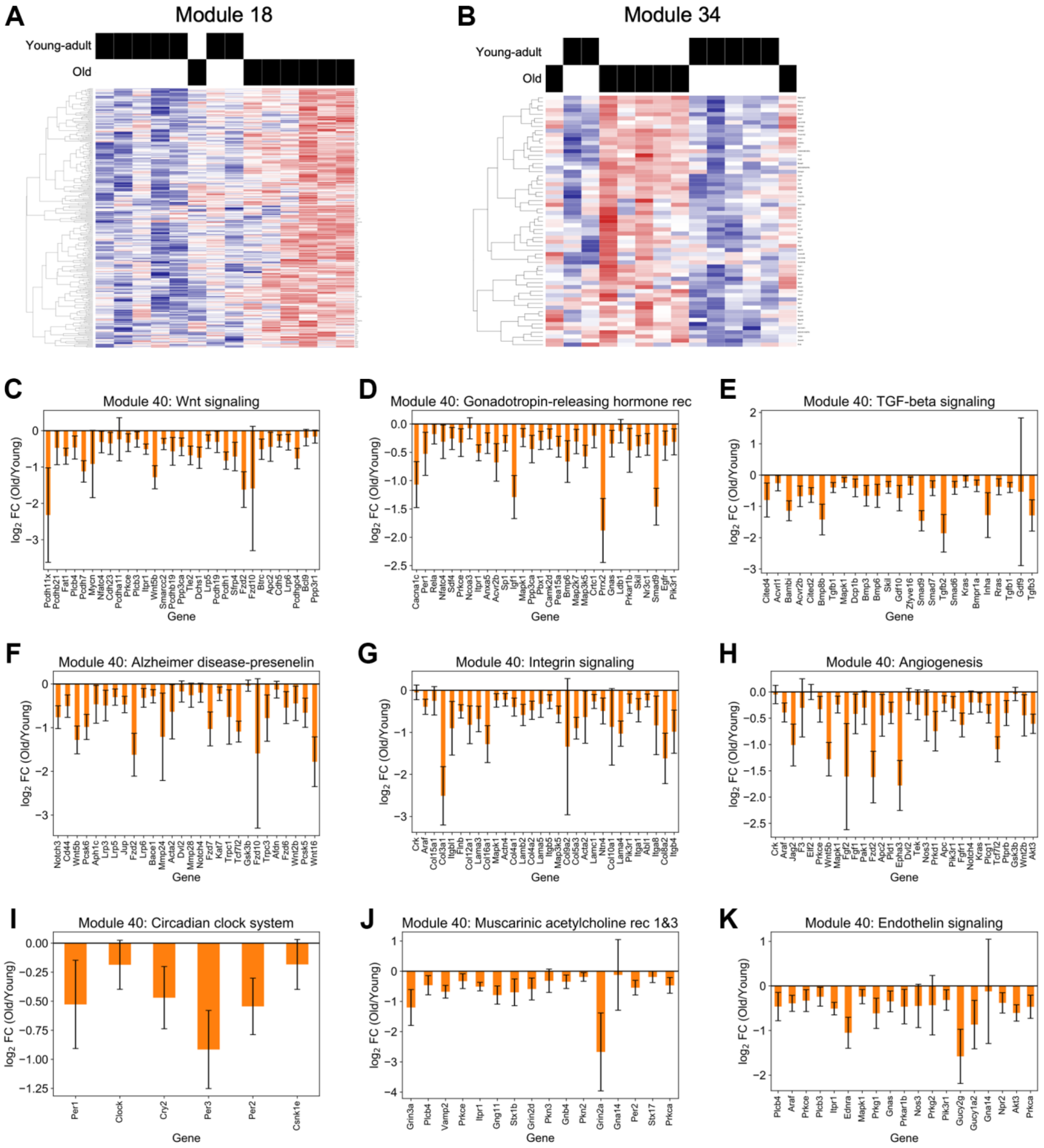

Supplementary Figure 2. WGCNA Supplement (**A**, **B**) Modules 18 and 34 did not display perfect separation. (**C**–**K**) Module 40 enriched pathways were nearly all downregulated.

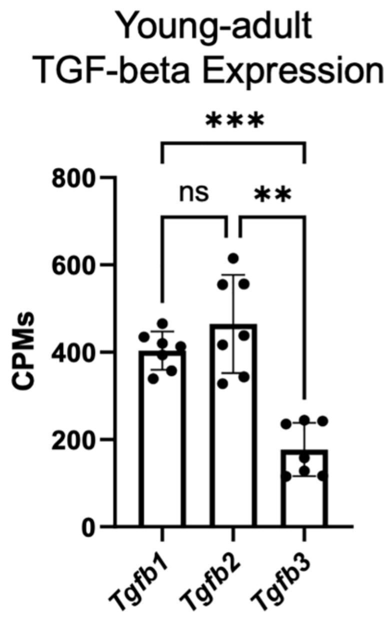

Supplementary Figure 3. TGF-beta gene expression from RNAseq CPM data (counts per million, CPMs) of Tgfb transcripts from young-adult bone. This complements the old vs. young fold-change data for these transcripts shown in Figure 4E.

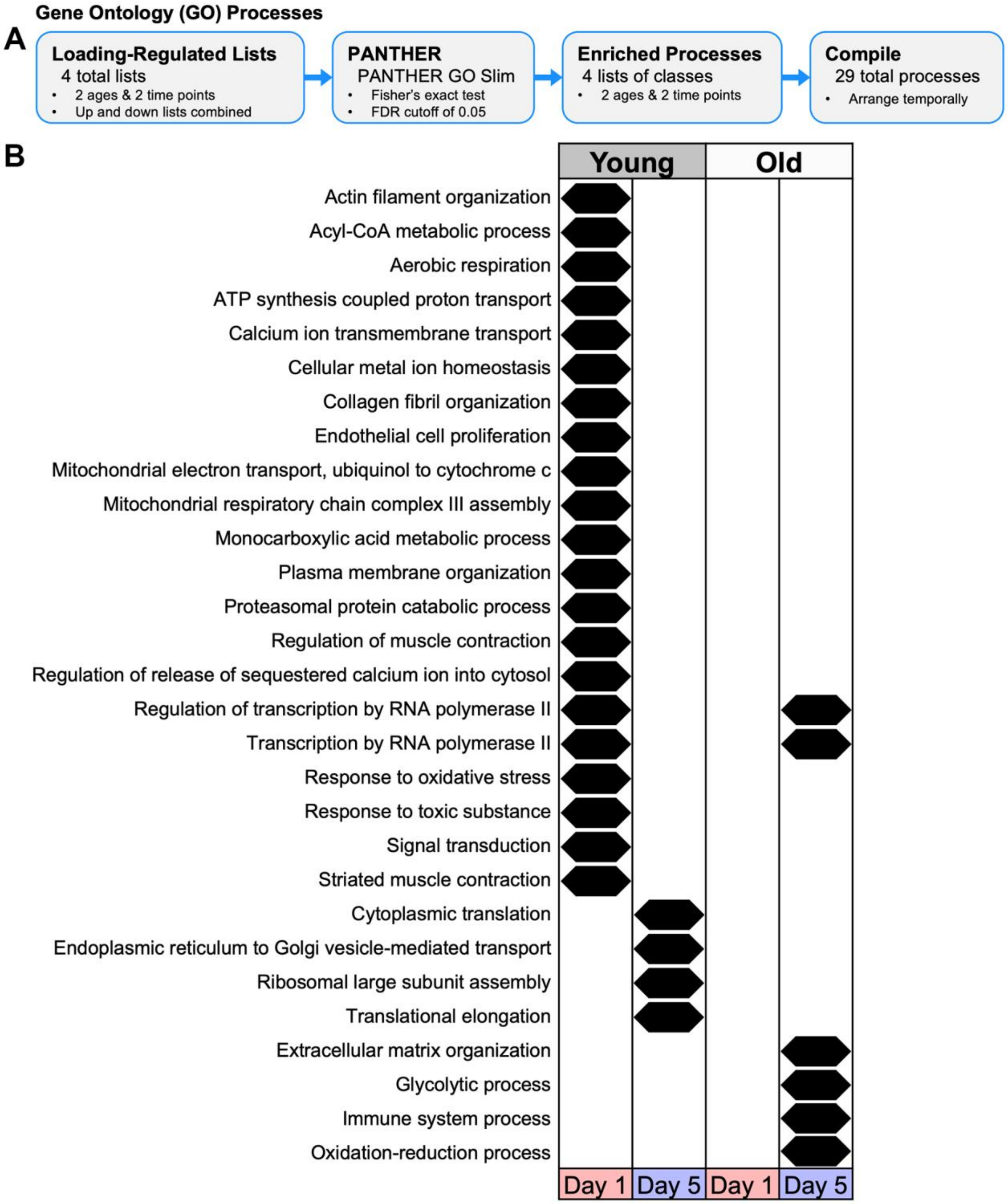

Supplementary Figure 4. Temporal map of PANTHER gene ontology analysis. (**A**) Loading-responsive protein lists for each age and day combination (up and down lists combined) were input to PANTHER GO Slim to identify enriched gene ontology processes. (**B**) PANTHER identified several enriched processes at each day and age combination.

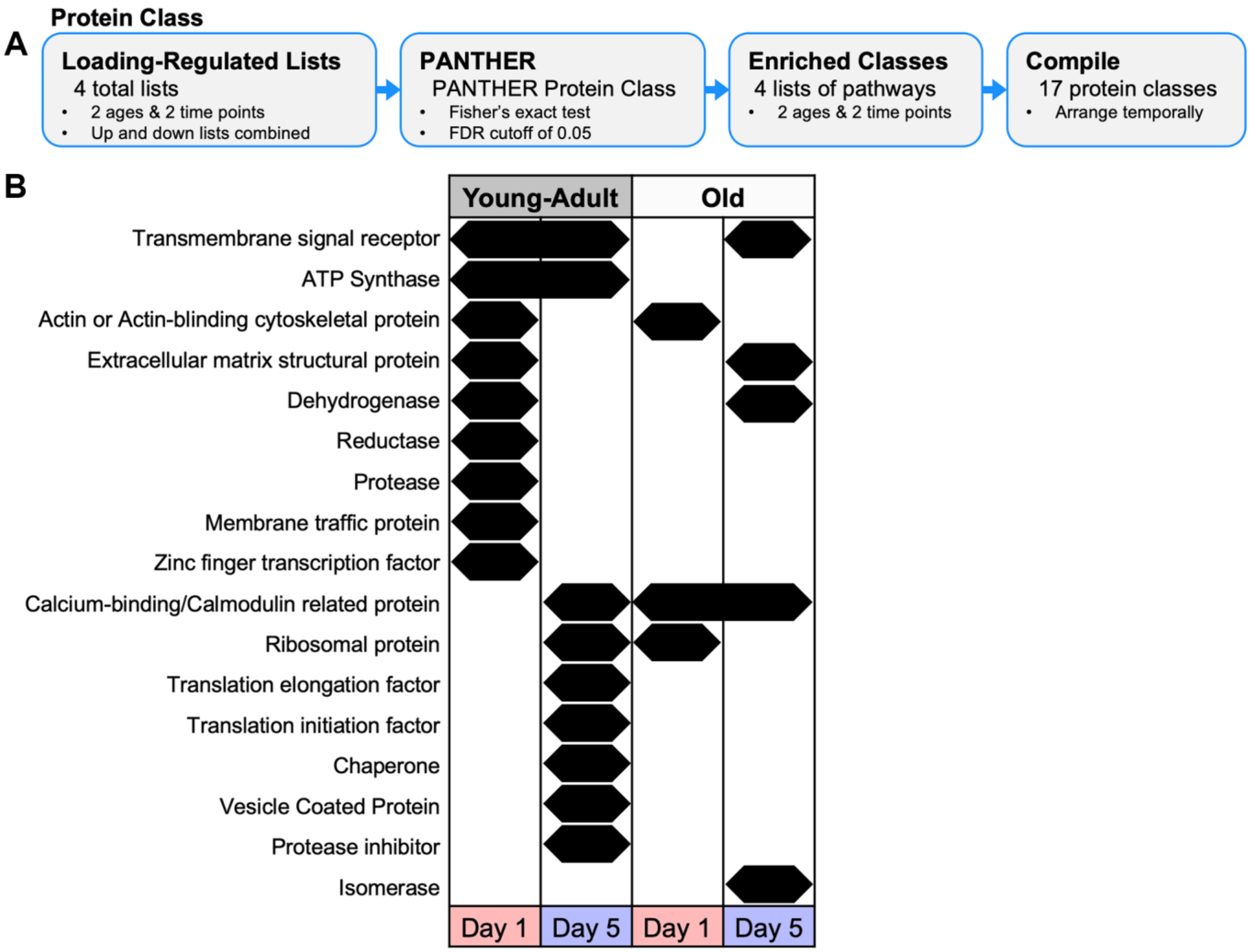

Supplementary Figure 5. Temporal map of PANTHER protein classes. (**A**) Loading-responsive protein lists for each age and day combination (up and down lists combined) were input to PANTHER to identify enriched protein classes. (**B**) PANTHER identified several enriched classes at each day and age combination.

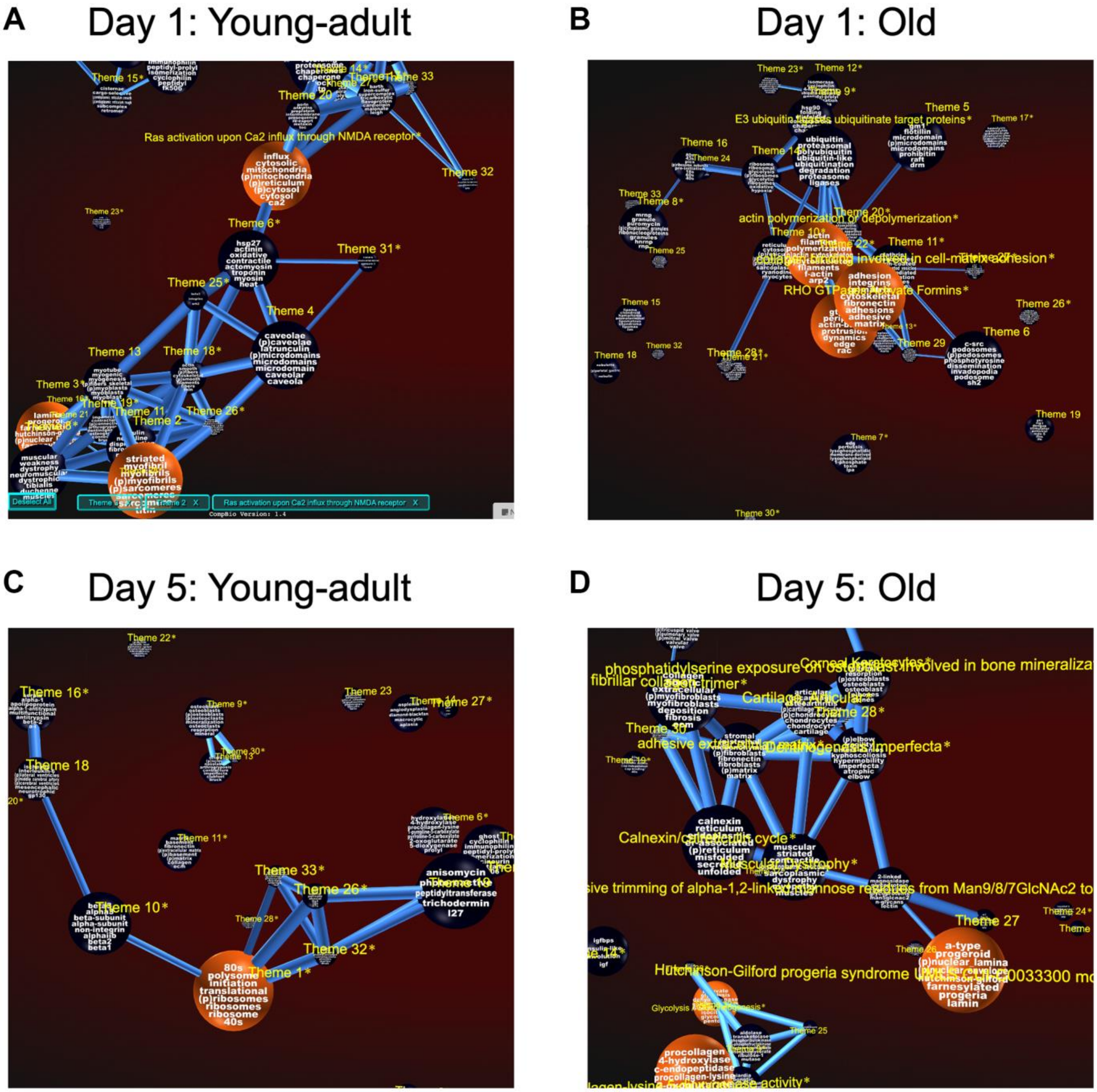

Supplementary Figure 6. COMPBIO loading analysis. (**A**–**D**) Loading-responsive protein lists for each age and day combination (up and down lists combined) were input to COMPBIO to identify enriched themes. COMPBIO identified numerous themes in each day and age combination.

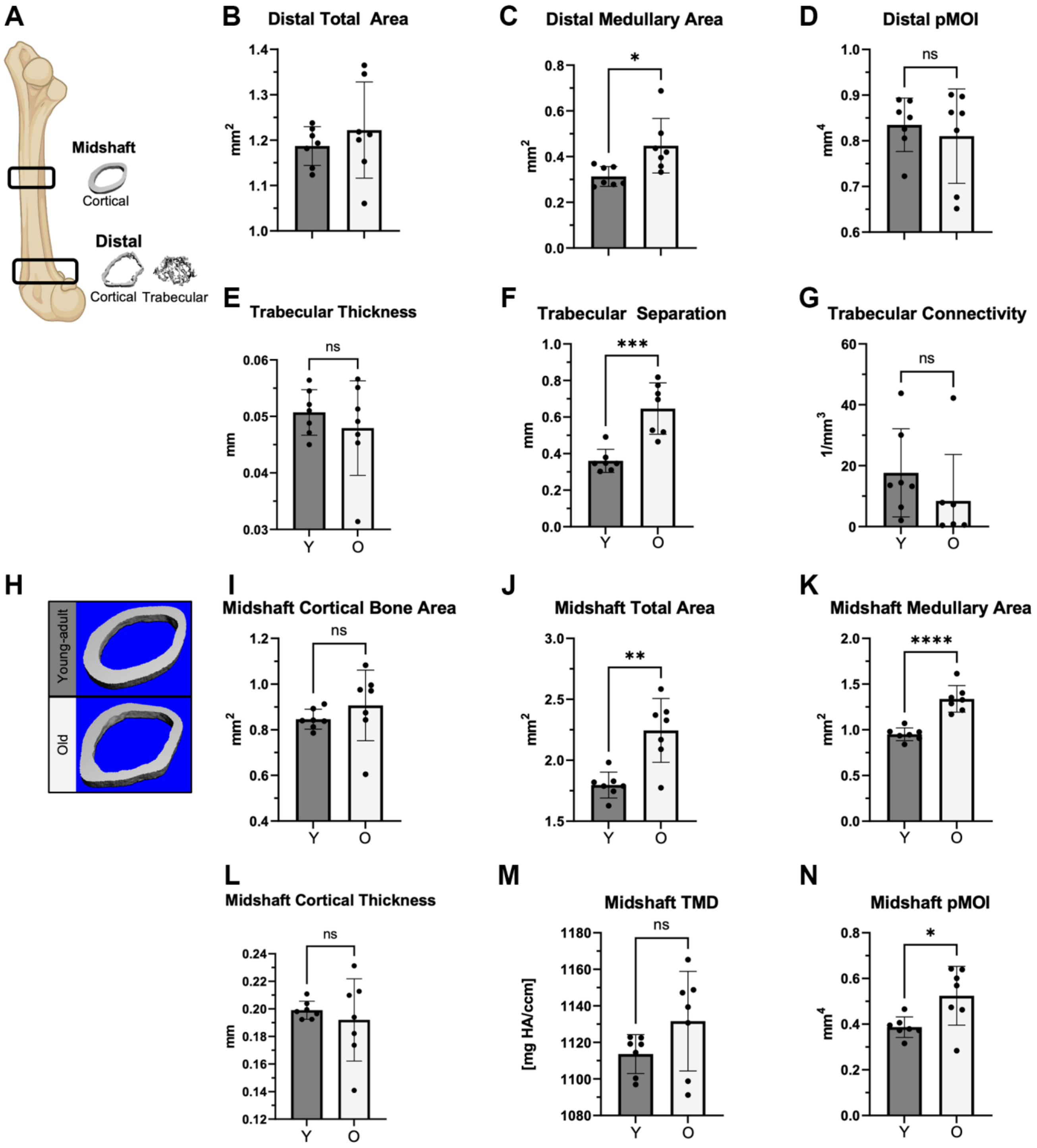

Supplementary Figure 7. Mice from the baseline study displayed the expected age-related differences and increased variation in bone properties by MicroCT. (**A**–**N**) Expected differences in bone properties were observed in the mice used in this study.

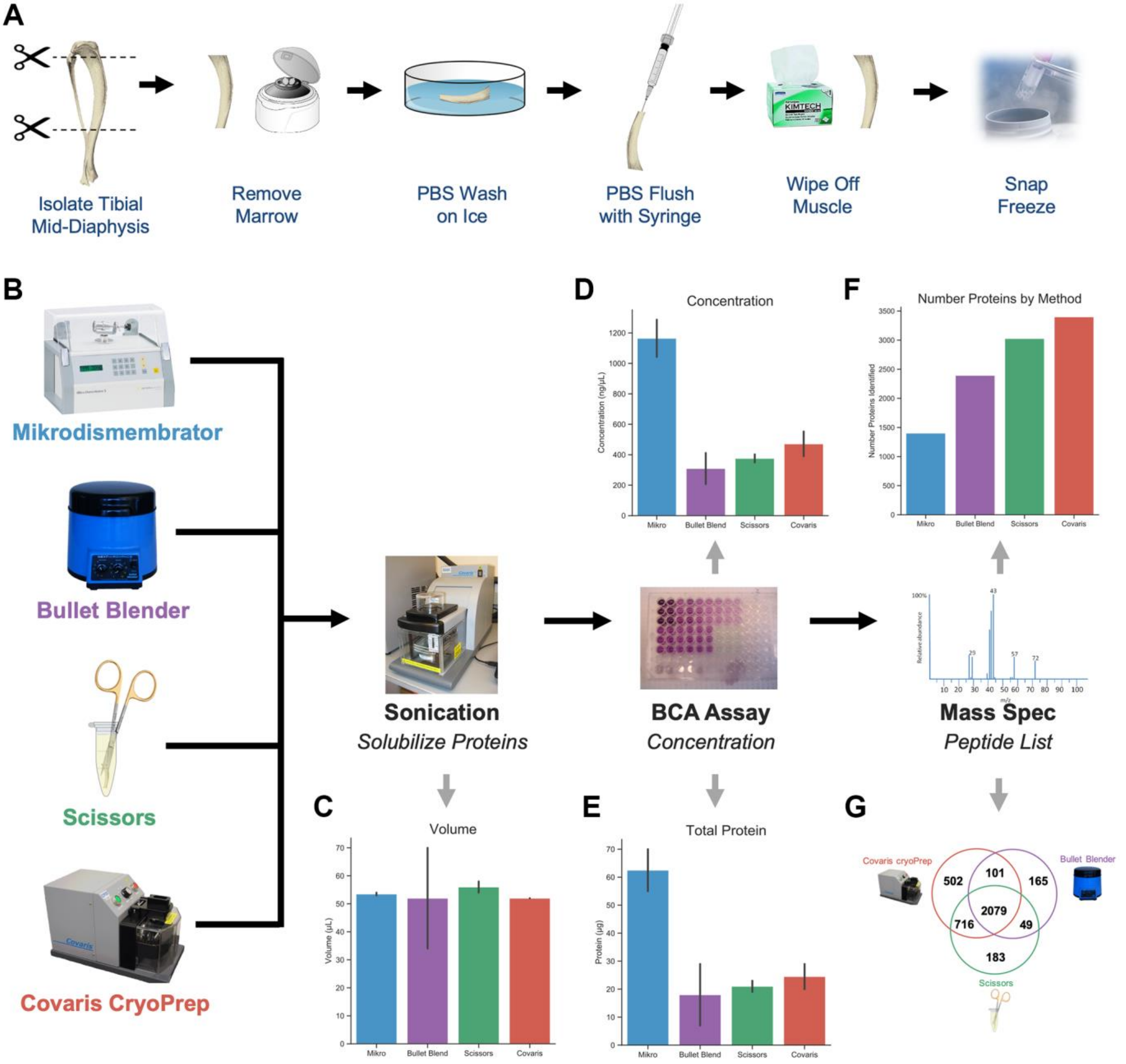

Supplementary Figure 8. Covaris CryoPREP was the optimal homogenization method for bone proteomics. (**A**, **B**) First, we used label-free proteomics to determine the optimal homogenization method for cortical bone from four commonly used methods in the bone field—the Mikrodismembrator (Braun), Bullet Blender (Next Advance), chopping with scissors, and the cryoPREP (Covaris). For each method (*n* = 2 young-adult samples per method), we used a BCA assay to measure the protein mass yielded and label-free proteomics to determine the number of proteins identified. (**C**–**G**) We found that while the MikroDismembrator yielded the greatest mass of protein relative to the other methods, it identified the fewest unique proteins. In contrast, the cryoPREP, which yielded the second highest mass of protein, identified the highest number of unique proteins. In fact, the cryoPREP identified more than double the number of unique proteins compared to the MikroDismembrator. For the remaining experiments, we proceeded using the cryoPREP.

A

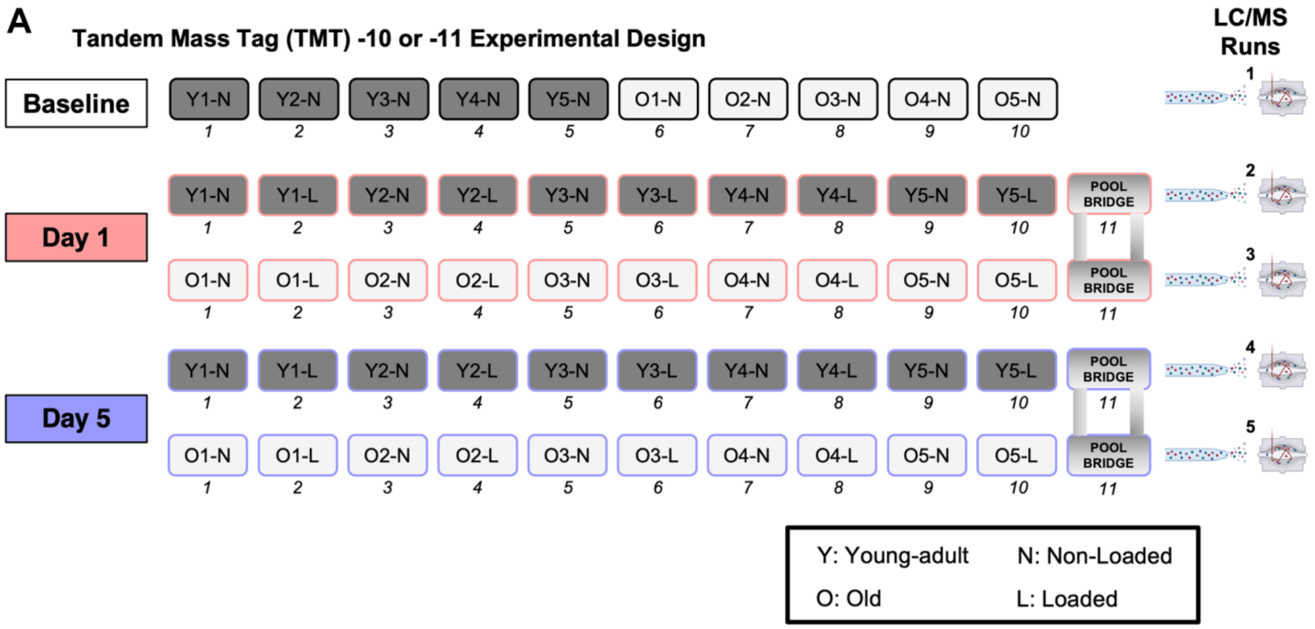

B **Total Mice Used**

EXPERIMENT	Young-adult	Old	Total
Paired Loading RNAseq/Proteomics	7	7	14
RNAscope & Histology	7	7	14
Day 1 Loading (TMT)	5	5	10
Day 5 Loading (TMT)	5	5	10
Pulverization Methods (LFQ)	10	-	10
**TOTAL**	**34**	**24**	**58**

LFQ: Label-free quantification

Supplementary Figure 9. Tandem Mass Tag-11 (TMT-11) experimental design and mice used across all experiments. (**A**) Tandem Mass Tag-11 experimental design for age and loading status. (**B**) Mice used across experiments.

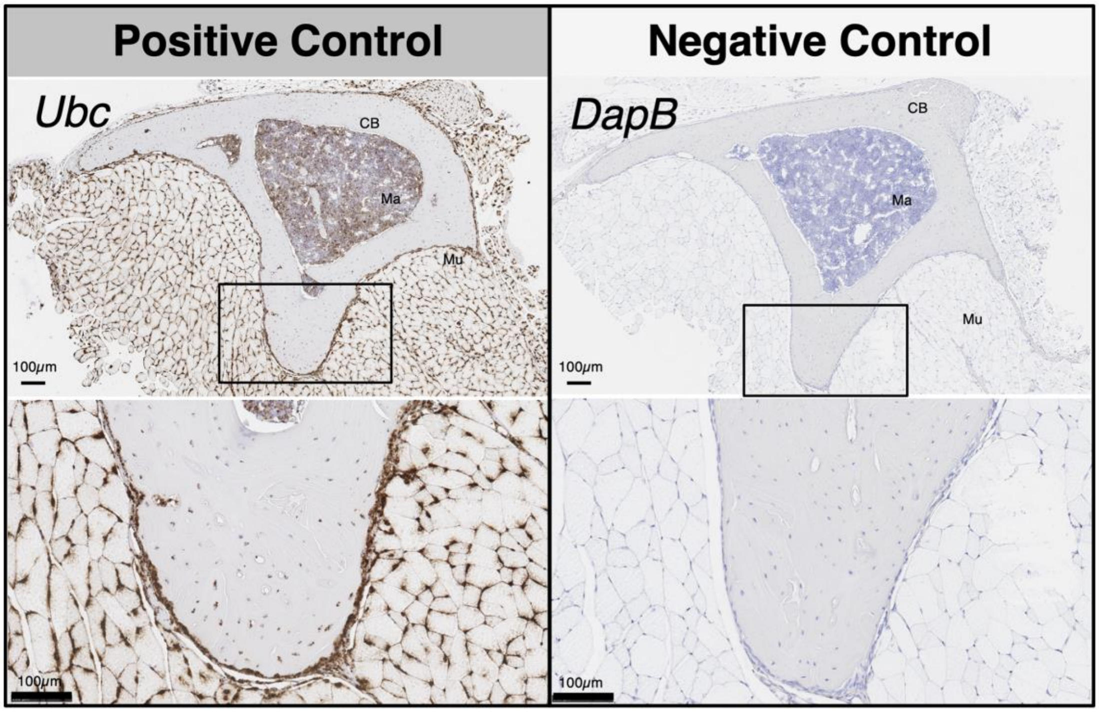

Supplementary Figure 10. Representative RNA ISH positive and negative controls. RNA ISH positive (Ubc) and negative (DapB) controls. These are representative images of the controls for all RNA ISH probes, i.e., they represent the controls for probes shown in Figs 2G (Asrgl1), 2L (Timp2) and 4G (Tgfb2). For each batch of RNA ISH, we ran a single positive and negative control sample. These control samples were selected to include one young-adult and one old sample per run. However, because of the limited number of serial sections available from each sample and the expense of the RNA ISH reagents, we did not run controls of all samples in each run, thus we cannot match 1:1 the images shown in Figures 2G, 2L and 4G with the controls shown.

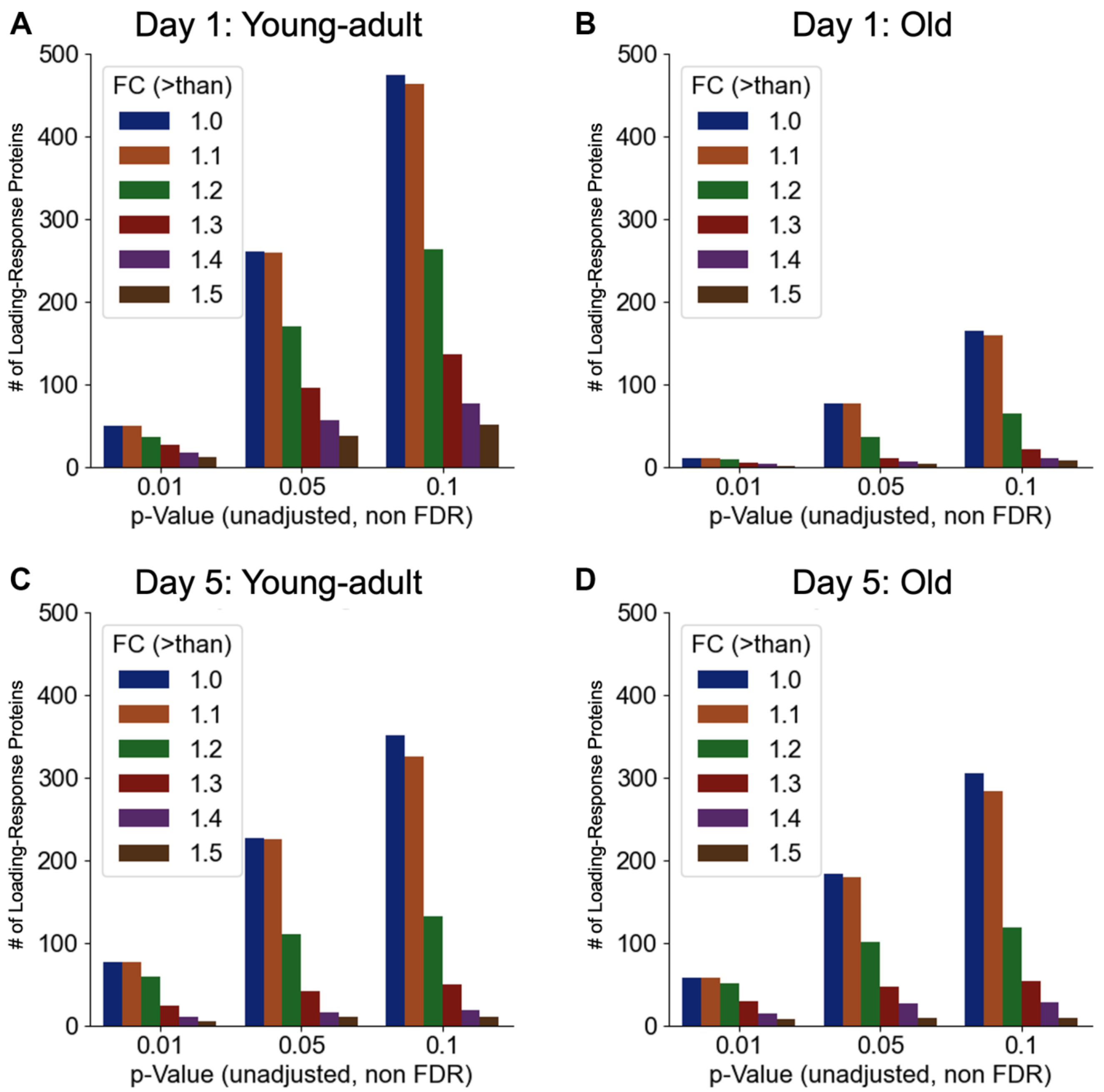

Supplementary Figure 11. Sensitivity analysis of the criteria for defining loading-responsive proteins. (**A**) Day 1 Young-adult. (**B**) Day 1 Old. (**C**) Day 5 Young-adult. (**D**) Day 5 Old. A cutoff of FC >1.1 (up or down) and unadjusted *p*-value < 0.05 was used to identify a reasonable number of proteins to facilitate downstream analysis.
