# Supplementary material for: A proteomics approach to study mouse long bones: examining baseline differences and mechanical loading-induced bone formation in young-adult and old mice
Source: Aging (Albany NY). 2024 Oct 12;16(19):12726–68. doi: 10.18632/aging.206131 (PMC11501390; doi:10.18632/aging.206131)
Supplement: Supplementary Table 1 [file aging-16-206131-s002.pdf]

SUPPLEMENTARY TABLE

Supplementary Table 1. RNAscope probes used.

Target	Catalog #	Notes
Timp2	567831	–
Asrgl1	1076841-C1	20ZZ probe custom designed to target Mm-Asrgl1 (256-1215 of NM_025610.3)
Tgfb2	406181	–
Pos Control*: Ubc*	310771	–
Neg Control*: DapB*	310043	–
